# Learning to dispatch volunteers to out-of-hospital cardiac arrests

**DOI:** 10.1007/s10729-026-09763-9

**Published:** 2026-05-28

**Authors:** Pieter L. van den Berg, Océane Fourmentraux, Shane G. Henderson, Caroline J. Jagtenberg, Hemeng Li

**Affiliations:** 1 Rotterdam School of Management, Erasmus University Rotterdam, Burgemeester Oudlaan 50, 3062 PA Rotterdam, the Netherlands; 2 School of Operations Research and Information Engineering, Cornell University, Ithaca, NY USA; 3 School of Business and Economics, Vrije Universiteit, Amsterdam, the Netherlands

**Keywords:** Community first response, Dispatch, Emergency Medical Services, Multi-class classification, Dynamic programming

## Abstract

Survival for out-of-hospital cardiac arrest can be significantly improved through volunteer efforts. To shorten the time to good-quality cardiopulmonary resuscitation, some emergency call centers use mobile phone technology to rapidly locate and alert nearby trained volunteers. Some such community first responder systems use phased alerts: notifying increasingly many volunteers with built-in time delays. The policy that defines the phasing of alerts affects both response times, which have a direct relation to survival, and the burden on volunteers. We aim to optimize this policy, which involves trading off these two metrics. The policy may depend on real-time information: where the volunteers are observed in relation to the patient and how long triage took. A direct approach using dynamic programming yields some insights, but is too slow for real-time use. Our contribution lies in recasting this problem as a multi-class classification problem and solving it using empirical data from Auckland, New Zealand’s community first response system. This case study shows that phasing the alerts based on real-time information provides important improvements relative to a competitive baseline that is indicative of current practice.

## Introduction

Certain medical emergencies, such as out-of-hospital cardiac arrests (OHCAs), require urgent medical attention, and rapid initiation of treatment can significantly increase survival rates. Many countries, including 19 out of 29 in Europe [23], have adopted a so-called community first responder (CFR) system. GoodSAM and Pulsepoint are two such systems introduced in multiple countries, including the UK, New Zealand, and the USA. CFRs are volunteers with some level of medical training who are willing to help during emergencies. CFR systems complement traditional ambulance services, ensuring timely response.

**Fig. 1:**
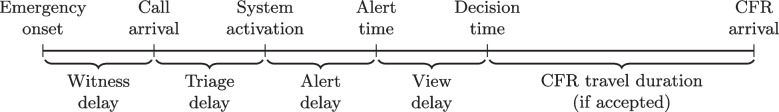
CFR response process

CFRs install an application on their smartphone that tracks, through GPS, their location and shares that location information with an application running in the emergency call center. When a medical emergency arises, the emergency call center can identify registered CFRs who are close to the medical emergency, and alert selected CFRs via the smartphone application. Besides CFRs, ambulances are dispatched as usual. While ambulances may be expected to follow instructions, alerted CFRs are not obligated to respond. However, those who choose to do so can proceed to the scene and provide first aid within the scope of their training until the independently dispatched ambulance crew arrives to take over. The central goal is to reduce morbidity and mortality by reducing response times. The CFR response process is summarized in Fig. 1. There, the view delay is the time from the volunteer receiving the alert to responding, which can be indefinitely long if the volunteer chooses to ignore the alert.

Systems such as GoodSAM cope with inherent uncertainty regarding whether CFRs will accept their alert. From the perspective of a single patient, it is desirable to notify *all* nearby CFRs to minimize the arrival time of the first-arriving responder. However, this strategy may lead to volunteers receiving excessive alerts or an overwhelming number of volunteers arriving at the scene, both of which are believed to decrease the probability of volunteers accepting future alerts. This issue, known as volunteer fatigue, is a common challenge encountered in various volunteer schemes [19]. Thus, it is important to find a trade-off between the current CFR response time and the future CFR acceptance rate.

A dispatch strategy adopted by some CFR systems is to send *phased alerts*. Under this strategy, alerts are sent in batches with time lags in between, and once an acceptance is received from a CFR, future batches are not sent. The party managing GoodSAM in New Zealand, ambulance provider Hato Hone St John New Zealand, uses batches of three alerts and a 30s time lag between batches. While reasonable, this strategy does not explicitly consider volunteer fatigue. Moreover, the batch sizes and time lags are fixed and do not take into account the real-time locations of nearby CFRs, which are known at the time of the incident. To the best of our knowledge, to date, there are no CFR systems that use situation-dependent dispatch rules.

In contrast to current practice, we present methods for determining which volunteers to alert and when for each incident, effectively balancing response times and volunteer fatigue and using real-time information on CFRs’ locations and dispatched ambulances. We first introduce a Markov Decision Process (MDP) formulation that maximizes an objective function combining survival probability and volunteer fatigue. This objective function depends on a parameter that determines the tradeoff between survival and volunteer fatigue. Such tradeoffs are inevitable in this multi-criteria decision problem. We then analyze the characteristics of MDP optimal policies. Recognizing the computational demands of obtaining an MDP optimal solution with increasing problem size, we offer an alternative data-driven approach using simulation and classification algorithms. This method can quickly identify the “best" policy among a set of pre-defined policies for each incident, facilitating real-time decision-making and easy deployment in practice. Additionally, the selection process of this approach is highly interpretable compared to the MDP optimal solution. These two methods complement each other well, as we can observe important characteristics from the MDP optimal solution and use those to construct our set of predefined policies for the selection strategy. We validate the performance of our proposed methods through a case study in Auckland, New Zealand. Finally, we consider the case where the desired balance between survival and volunteer fatigue is not specified in advance, and derive the efficient frontier.

The gains obtained through our data-driven approach may seem modest relative to current practice, but when measured in terms of lives saved are significant. Moreover, the gains are not uniformly spread over all incidents; some patients may receive a significant boost to their survival probability with our policies relative to current practice. The gains come with no additional cost relative to current practice – just a change in the alerting strategy.

The use of an Automated External Defibrillator (AED) has a significant impact on cardiac arrest survival. Therefore, New Zealand’s most active CFRs recently received their own ultra-portable AED and the effectiveness of this solution is being tested in a randomized controlled trial [31]. Importantly, the CFR system in Auckland does not include instructions for public-access AED pickups and instead directs volunteers directly to the patient. Hence, for most of the paper, we address a system in which CFRs do not use AEDs, which also helps communicate the main ideas in a transparent setting. We include a hypothetical case study in the appendix involving AEDs to show that the use of data-driven policies - though not MDPs due to computational demands - extends to that more complex setting.

To simplify the presentation, we assume that a CFR that accepts an alert does not subsequently drop out, which can happen in practice. This simplifies optimal policies, since if the closest available CFR accepts an alert then no further alerts are required. Moreover, we assume that a single CFR suffices on scene, though it can be beneficial to have two CFRs on scene so they can share the effort in administering CPR. It is straightforward to redefine the notion of redundant volunteers in this case.

The remainder of this paper is organized as follows. Section 2 provides a review of the related literature. Section 3 elaborates on the volunteer dispatch problem and introduces the MDP formulation. Section 4 presents a data-driven approach, based on simulating data and training a classification tree. In Sections 5 and 6, the application of our methods is demonstrated within the context of Auckland, New Zealand. We conclude with a discussion on the managerial implications of our findings and outline potential directions for future research in Section 7. An appendix contains proofs of several results, including some results that do not appear in the main paper. The appendix also contains numerical results that show, in some cases, even stronger results than those in the main paper, along with an example demonstrating that tree-based strategies can be obtained when explicitly modeling public-access AEDs.

## Literature

This section reviews the operations research literature on volunteer dispatch. Section 2.1 summarizes the literature specific to CFR systems for OHCA patients. Section 2.2 reviews studies on volunteer dispatch in other domains. Finally, Section 2.3 reviews work that inspires our proposed tree-based volunteer dispatch strategy.

### Community first response for OHCA

In recent years, an increasing number of papers focus on mathematical modeling of CFR systems. A common aim is to quantify the effectiveness of such systems, and methods include simulation [14, 15, 4, 27] and convex optimization [18]. These studies typically assume a predetermined dispatch strategy and vary another aspect, such as volunteer density or acceptance ratio. [18] develops a robust optimization formulation for positioning both drones that deliver AEDs and ambulances, taking into account the random response of CFRs; clever reformulation and solution techniques ensure the tractability of the resulting mixed integer programming model.

There are also studies that investigate how the dispatch process should be designed. In this context, a static approach entails optimizing a fixed dispatch radius [28]. Another approach is to focus on the time-dynamic aspect of the problem: alerts could be sent sequentially. [9] ask how this could be optimized. One way to study the sequential process is through discrete-event simulation, which allows the user to compare a handful of policies. This is done in [17], which evaluates three metrics: expected survival, number of alerts sent, and redundant arrivals. [17] formulate a dynamic program very similar to ours, though allow multiple classes of CFR that differ, e.g., in the probability that they will respond. As in our setting, solving the dynamic program is too slow for real-time application; [17] use model predictive control to obtain approximate solutions. They do not consider AEDs.

Some CFR systems include task differentiation: they prescribe which volunteer should pick up a public-access AED and who should travel directly to the patient. [20] attempts to optimize such task assignment. [22] and [21] expand on that effort with uncertain task compliance, where volunteers sometimes divert from their assigned task, either skipping or adding an unplanned AED pickup to their journey. While incorporating public access AED pickups seems sensible, it requires an extensive registry of AED locations including up-to-date maintenance records - something not every country can offer.

### Volunteer Dispatch in other Domains

Volunteer fatigue has been explored in many different volunteer schemes. [25] addressed the optimization of general volunteer labor assignments and showed that volunteer satisfaction is important for ensuring future participation. [19] were inspired by a platform that recovers food from local businesses and donates it to nonprofit agencies, crowdsourcing the transportation to volunteers. Similar to CFR systems, this platform alerts multiple volunteers in the hope that at least one will respond positively. They expect excessive notifications to reduce volunteer engagement and identify a trade-off between notifying more volunteers for the current task and preserving them for future ones. In contrast to our work, which focuses on dynamic alert policies considering real-time volunteer response, they formulated the corresponding online volunteer notification problem as a generalization of online stochastic bipartite matching, with the objective of minimizing the number of missed tasks. To capture a volunteer’s adverse reaction to excessive notifications, they assume that a notification triggers a random period of inactivity.

Other studies also explore volunteer dispatch policies through optimization. [2] discuss volunteer gleaners who harvest leftover crops donated by farmers. Their paper aims to increase operational effectiveness by better managing uncertain food and labor supplies. It optimizes a dynamic volunteer-staffing policy, specifying staffing levels for each crop type based on the number of available gleaners and the backlog of donations. [8] investigate the scheduling of a mixed workforce consisting of centrally scheduled paid staff and self-scheduled volunteers. By accounting for volunteers’ responses to changes in scheduling options, they reduce over-covered and under-covered time slots while keeping volunteers engaged.

Similar to CFR systems, spatial crowdsourcing involves tasks with assigned spatial locations to which workers or volunteers must travel [11]. In contrast to our work, which focuses on minimizing the time to complete a single task, most spatial crowdsourcing literature addresses a large number of tasks with goals such as task matching, quality control, incentive mechanism design, or privacy protection, as surveyed in [3]. One major difference that separates CFR systems from the aforementioned works is the extreme time sensitivity of volunteer dispatch for OHCA, where tasks must be completed within a very short period. Additionally, due to the infrequent occurrence of OHCA incidents, the sparsity of incidents in both time and space prevents us from formulating our dispatch problem as an online matching problem, leading us to focus on single-incident scenarios.

### Machine learning and policy selection for real-time decision making

In recent years, there has been significant work demonstrating the potential of using machine learning to support real-time decision-making. [3] explores the use of machine learning (ML) to support time slot management decisions in online grocery delivery. Their primary focus is on dynamically checking the feasibility of inserting a new customer order into existing delivery schedules. They transformed the feasibility check from solving a complex vehicle routing problem with time windows into a binary classification problem.

There are also studies focused on adaptively selecting the best policy among a finite set of alternatives. [3] considered the use of classification trees in this context due to their high interpretability. A related strand of work is contextual ranking and selection [1, 5, 13, 7, 12], which provides a method to determine an estimated best policy from a predefined set based on training using extensive offline simulation. Our work incorporates aspects of both approaches by using simulation to evaluate each policy among the set of alternatives, then formulating the real-time decision as a classification problem using decision trees.

## Markov Decision Process Formulation

In this section, we present a finite-horizon MDP formulation of the volunteer dispatch problem in discrete time with fixed time increments. Let $\mathcal {N}=\{1, \dots , N\}$ denote the set of volunteers within the dispatch radius at the time of volunteer system activation, ordered by their travel time, from shortest to longest. We assume a single ambulance is dispatched and that the travel times for both the volunteers within the dispatch radius and the dispatched ambulance are known and deterministic at the time of volunteer system activation. Let *T* be the number of time steps it takes for the dispatched ambulance to reach the patient, and $D_n$ be the number of time steps for the *n*-th closest volunteer. Without loss of generality, we consider only volunteers with $D_n \le T$ since there is no point in dispatching volunteers that would, even if they were to depart immediately, still arrive after the ambulance. The volunteer system activates at time step $t=0$, and the planning horizon ends when the ambulance reaches the patient at $t=T$.

### State Space

The state space consists of three parts: *t*, $\mathbf {v_t} = \{v_t^1, \dots , v_t^N\}$ and $r_t$. Here, *t* represents the current time step. Each $v_t^n, n\in \mathcal {N}$ provides the status of each volunteer at *t*. The term $r_t$ indicates the number of time steps remaining for the current fastest responder to reach the patient, whether the fastest responder is a volunteer or the dispatched ambulance.

The status of each volunteer, $v_t^n$, can be one of four possible states: 0, 1, 2, or 3, where 0 indicates that the volunteer has not been alerted; 1 indicates that the volunteer has been alerted but has not yet responded; 2 indicates that the volunteer has been alerted and has accepted the alert; and 3 indicates that the volunteer has been alerted and has rejected the alert. This choice of state space does not capture the time that has passed since each volunteer was alerted, which is necessary for tractability, but has the drawback that view times are modeled as memoryless.

At the time of system activation no volunteers have been alerted, so $v_0^i = 0$, for all *i*. Consequently, $r_0 = T$, indicating that the dispatched ambulance is the fastest responder at the moment. States with $r_t = 0$ are considered terminal states because they indicate that the first responder has already arrived at the scene, and no further actions are needed.

### Action space

Under the assumption of homogeneous volunteers, we focus on policies that prioritize alerting the nearest volunteer first. A simple interchange argument shows that this approach is optimal, because alerting the closer volunteer increases the chances of an early arrival and, thus, the survival rate. We further constrain ourselves to policies that permit multiple alerts to be sent out only at the volunteer system activation ($t=0$), with at most one alert sent at each subsequent time step. At the time of system activation, the action space is thus given by $A_0 = \{0, 1, \dots , N\}$, indicating how many volunteers to alert initially. For each subsequent time step, the action space is $A_t = \{0, 1\}$, where 0 indicates that no additional alerts are issued, and 1 indicates that the next closest unalerted volunteer should be alerted. While one can imagine alerting multiple volunteers at any single time point, with sufficiently small time steps there is little loss in performance and a gain in computational tractability.

### Transition

Let $s_t$ be the state at time *t*. We denote $p(s_{t+1} | s_t, a_t)$ as the probability of transitioning from $s_t$ to $s_{t+1}$ following action $a_t$. The evolution of state $s_t$ follows the action $a_t$ and is influenced by a random element $\omega (s_t, a_t)$ that characterizes volunteer responses. If $a_t=0$, the status of all unalerted volunteers remains unchanged. If $a_t=1$ and the next closest unalerted volunteer is *n*, then $v_{t+1}^n$ transitions from 0 to 1, indicating that the volunteer has been alerted. Each alerted volunteer who is yet to respond has a fixed probability $p_a$ of accepting the alert and $p_b$ of rejecting it during each time step, with $1-p_a - p_b$ being the probability of no response from the volunteer during the time step. Once a volunteer accepts or rejects an alert, their status does not change further and the residual time until the first responder arrives on scene, $r_t$, is updated accordingly.

### Reward function

We maximize the overall effectiveness of volunteer dispatch, which is modeled as the survival probability of the patient subtracting the volunteer fatigue. For systems like the GoodSAM operation in New Zealand, volunteers receive, on average, 2 or 3 alerts a year, so it seems unlikely that the number of alerts is a cause of fatigue. We instead model volunteer fatigue as being proportional to the number of redundant volunteers. We consider those volunteers arriving after the first responder, whether ambulance or volunteer, has already reached the scene as redundant. This modeling choice is intended to capture volunteer frustration in the event their skills are not required. Given that CPR can be tiring, one might instead define redundant volunteers to be those arriving after the ambulance or after the *second* volunteer. We did not do that in the present paper, but it would be straightforward to implement. The system only incurs a reward upon entering terminal states, i.e., when the first responder has reached the patient. To define those rewards, denote *f*(*s*) as the expected survival probability and *g*(*s*) as the expected number of redundant volunteers upon entering terminal state *s*. We quantify patient survival following [36], which was fit on data where both ambulances and volunteers responded, and volunteers did not carry defibrillators. This function takes the time to CPR (the time of first response, whether by volunteer or ambulance) and the time to EMS (time of arrival of the ambulance) from OHCA onset in minutes as inputs, and is given by

1 $$\begin{aligned} \phi (t_{CPR}, t_{EMS}) = \left( 1 + \exp \{0.04 + {0.3} t_{CPR} + 0.14(t_{EMS}-t_{CPR})\} \right) ^{-1}. \end{aligned}$$

We use this function to compute *f*(*s*).

Let $\gamma$ represent a factor trading off survival and redundant volunteers that has, as units, survival probability per redundant volunteer, and let $h(s_t, a_t, s_{t+1})$ denote the reward for transitioning from state $s_t$ to $s_{t+1}$ following action $a_t$. Then

2 $$\begin{aligned} h(s_t, a_t, s_{t+1}) = {\left\{ \begin{array}{ll} f(s_{t+1}) - \gamma g(s_{t+1}) & r_{t+1}=0 \\ 0 & \text {otherwise} \end{array}\right. }. \end{aligned}$$

We assume that all outstanding alerts without any responses are considered canceled 15 minutes after system activation. Consequently, only those volunteers who accept alerts within 15 minutes of system activation contribute to the count of redundant volunteers. Volunteers who arrive simultaneously with the ambulance are also counted as redundant.

### Optimal dispatch policy

Let $\tau$ denote the random time representing the arrival of the first responder, defined as $\tau = \min \{t\ge 0:r_t = 0\}$. For all $t = 0, 1, \ldots , T-1$ and states $s_t \in \mathcal {S}$, the value function following a policy $\pi$ is given by

3 $$\begin{aligned} V^\pi (s_t)&= \mathbb {E}_\pi \left[ \sum _{t=t}^{\tau -1} h(s_t, \pi (s_t), s_{t+1}) \big | s_t \right] \end{aligned}$$

4 $$\begin{aligned}&= \sum _{s_{t+1}} p(s_{t+1} | s_t, \pi (s_t)) \left[ h(s_t, \pi (s_t), s_{t+1}) + V^\pi (s_{t+1}) \right] . \end{aligned}$$

We determine the optimal volunteer dispatch strategy $\pi ^*$ by numerically solving the Bellman optimality equation

5 $$\begin{aligned} \pi ^*(s_t) = \text {argmax}_{a\in A_t} \left\{ \sum _{s_{t+1}} p(s_{t+1} | s_t, a) \left[ h(s_t,a, s_{t+1}) + V^{\pi ^*}(s_{t+1}) \right] \right\} \end{aligned}$$

using backward recursion. The numerical solution is rendered more efficient by exploiting properties of the optimal value function and policy that we explore next. Proofs are provided in Appendix A.

Our model involves a trade-off between the two objectives patient survival and the number of redundant volunteers. Proposition 3.1 demonstrates that the Pareto-optimal frontier between these two objectives is concave, indicating that each additional redundant volunteer on-scene yields a progressively smaller increase in patient survival.

#### Proposition 3.1

The Pareto-optimal frontier is concave with respect to the expected volunteer fatigue for any fixed OHCA.

Proposition 3.2 helps define a natural stopping time for the alerting process.

#### Proposition 3.2

Once someone has accepted, the optimal policy $\pi ^*$ will not send out any additional alerts.

Our next result formalizes the intuitive notion that at some point it is optimal to no longer alert volunteers, because they are so far away that they bring little additional survival probability benefits and are very likely to become redundant arrivals even if they respond. We first need some definitions relating to the benefit an alerted volunteer provides, along with their chance of becoming redundant. Let *s*(*d*, *t*) denote the expected increase in survival probability compared to that obtained solely from the ambulance response, assuming that there is only one volunteer, the volunteer is *d* time steps from the patient and is alerted at time *t*. Also, let *r*(*d*, *t*) denote the probability that such a volunteer arrives after the ambulance if the volunteer is alerted at time *t*.

#### Definition 3.3

Let *d*(*t*) be the minimum number of travel time steps such that any volunteers located beyond this threshold have an expected benefit that is less than the expected penalty, i.e.,

6 $$\begin{aligned} d(t) = \min \{ d\in \{0, 1, \dots , T\} :s(d,t) < \gamma r(d, t)\}. \end{aligned}$$

Proposition 3.4 proves that no alerts will be sent to volunteers whose travel times to the patient exceed a threshold.

#### Proposition 3.4

Suppose the *i*-th volunteer, located $D_i$ time steps away from the patient, is the closest unalerted volunteer. The optimal policy $\pi ^*$ will not alert this volunteer at time *t* if $D_i \ge d(t)$.

While the policies we obtain by numerically solving the MDP model are optimal, the running times are too long to be useful in real-time application for OHCAs. Moreover, the optimality of these solutions should always be considered in the context of the MDP assumptions, most notably the memoryless view times. Additionally, these policies can be difficult to interpret. Therefore, in the next section, we introduce an alternative data-driven method that is fast in real time, interpretable, and does not require the assumption of memoryless view times. The structural policies of the optimal policy derived above are helpful in setting up this data-driven method.

## Selection strategy using a classification tree

In this section, we approach the volunteer dispatch problem in a data-driven way, utilizing both simulation and classification algorithms. First, we introduce a finite set of predefined policies, each of which is considered potentially effective, at least for some incidents. In choosing these policies we are partially guided by the structural results for optimal policies in the previous section. Next, we describe a simulation model used to evaluate the effectiveness of each policy. Finally, we outline a selection strategy using classification algorithms to quickly identify the “best” policy from the predefined set for each incident. The simulations and classification algorithm can be run in a preparatory phase, which can be computationally demanding, but the use of the resulting strategy in real time is very fast, thereby facilitating real-time decision-making and practical deployment. An additional benefit is that the resulting strategy is interpretable, making the policy selection process and selected policy more transparent and understandable than is the case when using dynamic programming.

### Dispatch strategies and pre-defined policies

We distinguish between *strategies* and *policies*. A *policy* defines how CFRs are dispatched for a single incident, while a *strategy* determines which policy to use for each incident. CFR systems generally apply a *static* strategy, meaning they use a fixed policy for all incidents, even though the policy can adapt based on real-time CFR responses. In contrast, we propose an *adaptive* strategy, which selects the appropriate policy at the time of dispatch based on real-time information, such as CFR locations, assigned ambulances, and volunteer system triage delays.

Our approach converts the problem into a multi-class classification problem that assigns a class to every incident. To achieve this, we first define a finite set of policies that will serve as the classes. Although computing the optimal policy from the MDP model is computationally intensive, we have identified key characteristics from the MDP optimal policy that we want to incorporate into these predefined policies.

A key characteristic is that no further alerts should be sent once a volunteer has accepted an alert. Additionally, we observed that if a rejection is received, the next alert should be sent immediately rather than waiting, and it is often unnecessary to alert additional volunteers if there has been no response. After discussions with people who currently operate CFR systems, we came up with the following alert policies, starting the time count from the moment of CFR system activation:

1. *Send all at time 0*: alert all available volunteers within the dispatch radius immediately.
2. *Send n at time 0*: Alert *n* volunteers immediately at time 0, and send no alerts thereafter.
3. *Keep n active until time t*: Alert *n* volunteers at time 0 and replace every incoming rejection with an additional alert. After time *t*, stop sending alerts.

It is not essential that policies prescribe mutually exclusive actions. For example,“Send all at time 0" and “Send *n* at time 0” perform the same action on an incident with *k* volunteers, if $k \le n$. We included both of these policies because, when $k > n$, it may be important to notify up to a certain number of volunteers immediately, but not too many to avoid redundancy.

Many different types of dispatch policies exist. Our approach is flexible and can accommodate various dispatch policies. However, for the purposes of this paper, we focus on this predefined set. These policies are intuitive, readily implemented and incorporate key characteristics observed from the DP optimal policy. In addition, the *Keep n active until time t* policies resemble the *phased alert* policies that are commonly used in CFR systems. These policies go beyond the previous policies in that they *maintain* a number of alerts to try to ensure response, but only up to the time threshold *t*, after which an alerted volunteer might be likely to become redundant. We believe that these policies are sufficiently robust and, when selected in an incident-specific manner, cover a reasonable range of strategies.

### Simulation model

We evaluate the performance of each dispatch policy using a discrete-event simulation model that includes the entire CFR response process:

1. CFR system activation: sending the initial batch of alerts.
2. Alerted CFR response: accepting, rejecting or ignoring the alert.
3. Issuing additional alerts if needed, following the specified policy.
4. CFR arrival on-scene to treat the patient if they arrive before the ambulance.
5. Ambulance arrival.

Upon CFR system activation, we assume the dispatcher can observe the required travel times of the assigned ambulance and each CFR within the dispatch radius, and the triage delay, i.e., how much time has passed since the call arrived.

We assume the CFR responses, including whether they will attend and their corresponding view delay, follow a known distribution $\mathcal {F}$. We used an empirical response distribution based on the data provided by Hato Hone St John Ambulance Service. More details on the dataset are included in Section 5.2.

If no CFRs are available, only the ambulance will respond. If multiple CFRs are available, we assume only the first-arriving CFR contributes to patient survival; all CFRs arriving after the first responder, whether the first responder was a CFR or the assigned ambulance, are considered redundant. Any outstanding alerts that receive no response are considered canceled 15 minutes after system activation, as they are no longer helpful for the patient.

### Machine learning pipeline

Given the set of predefined policies, we aim to develop an *adaptive* strategy that identifies the “best" policy in real time whenever an OHCA occurs, using observable information about the OHCA. While any machine learning classification algorithm could be used for this purpose, we use a classification tree, partly inspired by [3], to learn an interpretable, tree-based CFR dispatch strategy.

We define the CFR response time as the sum of the triage delay and the CFR travel time. The predictors we use for the classification tree include the number of CFRs within the dispatch radius, their corresponding response times, and the realized CFR system triage delay. We use CFR response times instead of CFR travel times as features because considering triage delays and travel times separately can be misleading and may result in unnecessary misclassification. For example, a long triage delay added to a short CFR travel time can still yield a fast response. Therefore, it is more important to focus on the overall response time.

When learning this selection strategy, we assume a fixed ambulance travel time across all instances in the training set for simplicity and more intuitive results. However, our approach can be extended to include real-time estimates of ambulance travel time as an additional feature. We generated 10,000 instances as our training dataset, labeling the optimal dispatch policy for each instance using the simulation model described above. Additionally, we generated 2,000 instances as our testing set. We generated instances rather than simply using data primarily because the data only includes information on volunteers who were alerted, and not on volunteers who were within range and could have been alerted but were not, but also because the dataset is not big enough to accurately distinguish policies. More details on the instance generation process are included in Section 5.3, and we present the numerical results in Section 6.

We next train a classification tree that predicts the best policy for each instance, using the Classification And Regression Tree (CART) algorithm. In our numerical work, we do this with the DecisionTreeClassifier from [26].

## Case description

Auckland is the largest city in New Zealand, with a population of 1.4 million within the greater Auckland region. Our study focuses on the most urban areas with a population density exceeding 1000 people per square kilometer. The focus area, with 1.1 million residents, is divided into 287 so-called area units. These area units are the second smallest unit at which Statistics New Zealand collects data [30].

### CFR response process

We follow the CFR response process as shown in Fig. 1. The time from call initiation to volunteer system activation is defined as the *triage delay*. The time from system activation to the issuing of the first set of alerts is defined to be the *alert delay* and is assumed to be zero. The time from the volunteer receiving the alert to responding is called the *view delay*, which can be indefinitely long.

We assume that accepting CFRs respond on foot, and use the most conservative estimate of [28] in the Netherlands to model their speed at 6 km/h, traveling as-the-crow-flies distances. We assume that CFRs are dispatched only if they are within 1 km of the patient and can reach the patient before the assigned ambulance, aligning with current practice. Consequently, the travel time for CFRs, assuming at least one is available within 1 km, is 10 minutes or less.

### Data

We used data provided by Hato Hone St John Ambulance Service (SJAS), which operates the GoodSAM app in New Zealand. The SJAS has provided us with all OHCAs from 2013 to 2020, with their locations and 911 call arrival times. The dataset also includes details of CFR alerts issued from inception of the program in 2017 to 2020, with the time and location of each alert, the CFRs’ responses (acceptance, rejection, or no response), and the arrival time at the scene based on GPS data.

Among the total 4157 OHCA incidents arising while the CFR system was operating and in the area of interest, 2827 had alerts sent to CFRs. Of the 6749 CFR alerts recorded, 698 were accepted, 263 were accepted but later dropped, 2026 were rejected, and 3762 received no response. If an alert was initially accepted but later dropped, it indicates that the CFR initially agreed to respond but later decided not to, possibly due to seeing someone else already assisting the patient or other reasons. We treat both outright acceptances and initial acceptances that are dropped later as acceptances, which leads to an empirical acceptance rate of 14%. We measured the triage delay from call arrival to the earliest alert time, and view delay from alert time to when CFRs make their decisions.

**Fig. 2:**
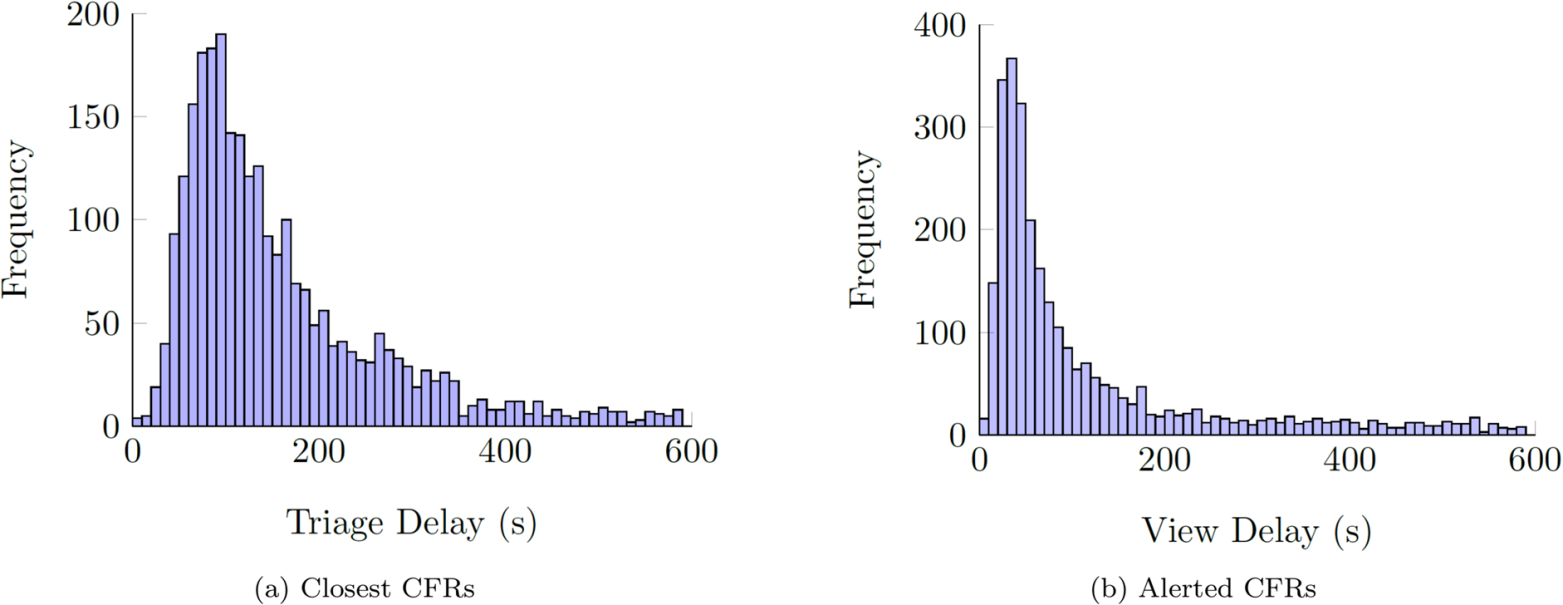
Left: Histogram of triage delays from the 2825 OHCA incidents in Auckland from December 2017 to December 2020. Right: Histogram of view delays from the 6749 alerts in Auckland from December 2017 to December 2020

Figure 2 presents the empirical distributions of triage delays and view delays. We omitted 2 incidents with missing call arrival times for the histogram of triage delays. The median triage delay is 2 minutes. Among all responding CFRs, 95% responded within 10 minutes, and 84% responded within 5 minutes. The peak view delay is between 20s and 50s, with the peak of those who accepted alerts responding slightly faster than those who rejected. This suggests that CFRs who accept alerts tend to respond more quickly. If a CFR did not respond, the decision time required to decide whether to accept or reject the alert is not recorded in the dataset. In this case, we assign an infinite view delay, which is not shown in Fig. 2b.

### OHCA incidents generation

In our discrete-event simulation, we sample the location of an OHCA using an area-unit specific incident rate that is estimated by integrating the SJAS dataset and demographics with socio-economic factors as discussed in [6]. One could instead use the empirical OHCA rate, but low counts in some area units have very high variability relative to their mean. More details and discussion on this estimation process can be found in Appendix 7 of [29].

We assumed that 0.1% of the population in each area unit are registered CFRs, which allowed us to calculate the volunteer density per area unit. We obtain the resident population in each area unit from the 2013 census [29], which we deemed representative for the OHCA data we received. We then applied the spatial Poisson point process model for CFR distribution as introduced in [36] to simulate the number and locations of volunteers within a 1 km dispatch radius. We could not have obtained this information from data because the data only records locations of volunteers who were alerted.

For each alerted volunteer, we bootstrap their view delay from the SJAS dataset. To preserve any potential dependence between the view delay and volunteer response (acceptance or rejection), we sample these variables concurrently. In addition, we sample the triage delay for each incident from the SJAS data by excluding any instances where the triage delay exceeds 10 minutes, as volunteer response with a delay of this length is unlikely to contribute to patient survival.

### Policies

We used the set of pre-defined policies discussed in Section 4. For *send n at time 0*, we consider the values of *n* between 0 and 10. We included “send 0 at time 0” as a policy because, with some incidents, all volunteers may be very far away from the OHCA. In such cases, volunteers will take a long time to reach the OHCA and therefore do little to increase survival, and also be very likely to be redundant due to the ambulance arriving first, so it may not be optimal to send any volunteers. Indeed, Send 0 is selected by the classification tree in some cases, which then allows a greater volunteer response in incidents where many volunteers are close to the OHCA. For *Keep n active until time t* we consider values of *n* between 1 and 10 and set *t* equal to 3, which proved most fruitful in extensive preliminary experiments. This gives a total of 22 policies.

### Performance metrics

To measure the effectiveness of each volunteer dispatch policy, we measure the patient survival and subtract the volunteer fatigue. We quantify the patient survival using (1), which requires volunteer and EMS response time measured from OHCA onset as input. In line with [36], we add a 1-minute delay from OHCA onset to call initiation. We obtain the CFR response time from call initiation from simulation. In our experimental design, we vary the ambulance response time deterministically to emphasize the different nature of dispatch decisions depending on the expected EMS response time. We consider three different ambulance response times from call initiation: a long response time of 12 minutes, which SJAS aims to meet for 95% of incidents; a short response time of 6 minutes, targeted for 50% of incidents; and a median response time of 9 minutes. Volunteer fatigue is calculated based on the number of redundant CFR dispatches, applying a multiplicative penalization factor of $\gamma =0.01$. This corresponds to equating 100 redundant arrivals to one survival.

## Numerical results

In this section, we present the performance of our proposed tree-based selection strategy through the case study described in Section 5. We first provide an example of the tree-based selection strategy in Section 6.1, then compare its performance with that of static strategies in Section 6.2. Recognizing that volunteer acceptance rates vary across different countries and regions, we examine the impact of the acceptance rate on dispatch policies in Section 6.3. Finally, we conclude the section with a comparison between the MDP optimal policy and the tree-based strategy in Section 6.4.

### High interpretability of tree-based strategy

A key advantage of the proposed tree-based selection strategy over the MDP formulation is its high interpretability, which is valuable in healthcare-related applications, including CFR systems.

**Fig. 3:**
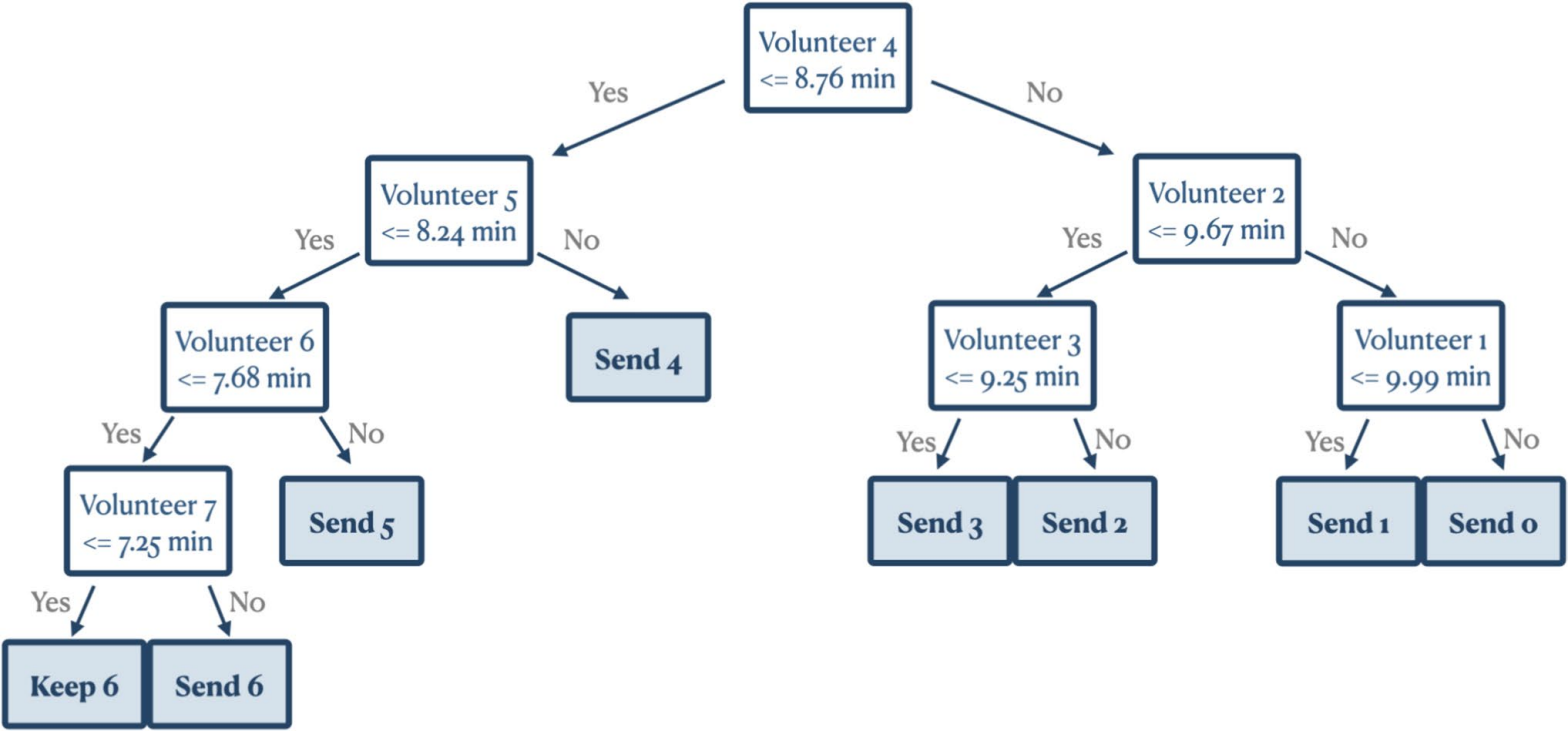
A tree-based selection strategy for instances in Auckland with 14% acceptance rate and 12-minute EMS response time measured from call arrival. Volunteers are numbered by how long they would take to reach the OHCA, with Volunteer 1 being the closest. So, for example, if there are 5 volunteers with response times (3, 5, 8, 9, 10) minutes, respectively, then the policy chosen is “Send 3.” To see why, starting at the top of the tree, the response time of Volunteer 4 is 9 minutes, which is *not* less than 8.76 min, so we proceed to the right branch of the tree. The response time of Volunteer 2 is 5 minutes, which is less than 9.67 min, so we proceed to the left branch. The response time of Volunteer 3 is 8 minutes, which is less than 9.25 min, so we proceed to the left branch, arriving at “Send 3.”

Figure 3 shows an example of the tree-based selection strategy trained on generated instances in Auckland with a 12-minute EMS response time, where each node represents a split in the feature space. For example, the first split is made based on the time it will take the fourth-closest volunteer to reach the patient. If that time is less than 8.76 minutes, we enter the left part of the tree. If it is more than 8.76 minutes, we enter the right part of the tree. The caption provides a worked example. The tree policy is intuitive, as it selects policies that send out more alerts when there are more CFRs within the dispatch radius and closer to the patient. Conversely, on the right side of the tree, with a smaller number of CFRs or when the closest CFRs are far away from the patient, the strategy refrains from sending out additional alerts. In addition, the tree-based selection strategy aligns with the characteristics of the MDP optimal policy, as stated in Proposition 3.4, by applying a threshold on volunteer travel times to decide on sending additional alerts.

The tree does not distinguish between low and high volunteer density areas. If there are *n* volunteers observed in the circle, it does not matter whether the original density from which the sample was drawn was high or low. Given our Poisson assumption, the distribution of distances will be the same. However, realizations with high volunteer numbers are more common in high-density areas. To test how this affects performance, we analyzed how the policy, that was generated for Auckland in its entirety, performs in the most rural (urban) part of Auckland. This yielded a survival of 0.0191 (0.0380), compared to 0.0267 city wide.

### Adaptive tree-based strategies vs. static strategies

We next compare the performance of the adaptive tree-based strategy with static strategies under three different EMS response times. We then focus on a 12-minute EMS response time, partly because SJAS aims to respond to 95% or more calls within 12 minutes. More examples of the tree-based strategies under different EMS response times are included in Appendix B.

Recall that our objective is defined as patient survival minus volunteer fatigue, with a penalization factor of 0.01, which has the interpretation of equating one additional survivor to 100 redundant volunteers. Table 1 provides the accuracy of our tree-based strategy on the testing set, measured as the fraction of test instances where the tree-based strategy correctly identified the “best" policy among the predefined set. We see a high accuracy when EMS takes 6 minutes to arrive, which drops to 91% accuracy when EMS takes 12 minutes to arrive. Even the latter is reasonably high, especially given the challenging nature of the multiclass classification problem with up to 22 classes. Moreover, many misclassifications arise due to very small differences in performance between the best policy and the one selected by the tree, that one might view as negligible, practically speaking; see Fig. 8. Table 1 also shows the average objective value, survival and number of redundant volunteers of the tree-based strategy compared to the best static strategy under each scenario. The best static strategy is defined as the policy from our pre-defined set that yields the highest average performance across all test cases when applied uniformly.

**Table 1:** Accuracy and average objectives reported by the tree-based selection strategy versus the average objectives of the best static strategy among our predefined policies under a 14% volunteer acceptance rate

EMS	Best static				Tree			
	Policy	Obj	Surv	Redundant	Obj	Surv	Redundant	Accuracy
6 min	Send 1 at time 0	0.1058	0.1067	0.0881	0.1068	0.1071	0.0306	0.993
9 min	Send 2 at time 0	0.0489	0.0501	0.1119	0.0503	0.0512	0.0871	0.964
12 min	Send 4 at time 0	0.0244	0.0263	0.1908	0.0253	0.0267	0.1380	0.911

We observed that as the EMS response time increases, the best static strategy issues more alerts. This is because shorter EMS response times provide less opportunity for CFRs to react, increasing the likelihood that CFRs will arrive after the ambulance and contribute to volunteer fatigue. As a result, shorter EMS response times often result in simpler dispatch policies being optimal, which leads to a simpler classification tree and higher accuracy in predicting the correct “optimal" strategy.

**Fig. 4:**
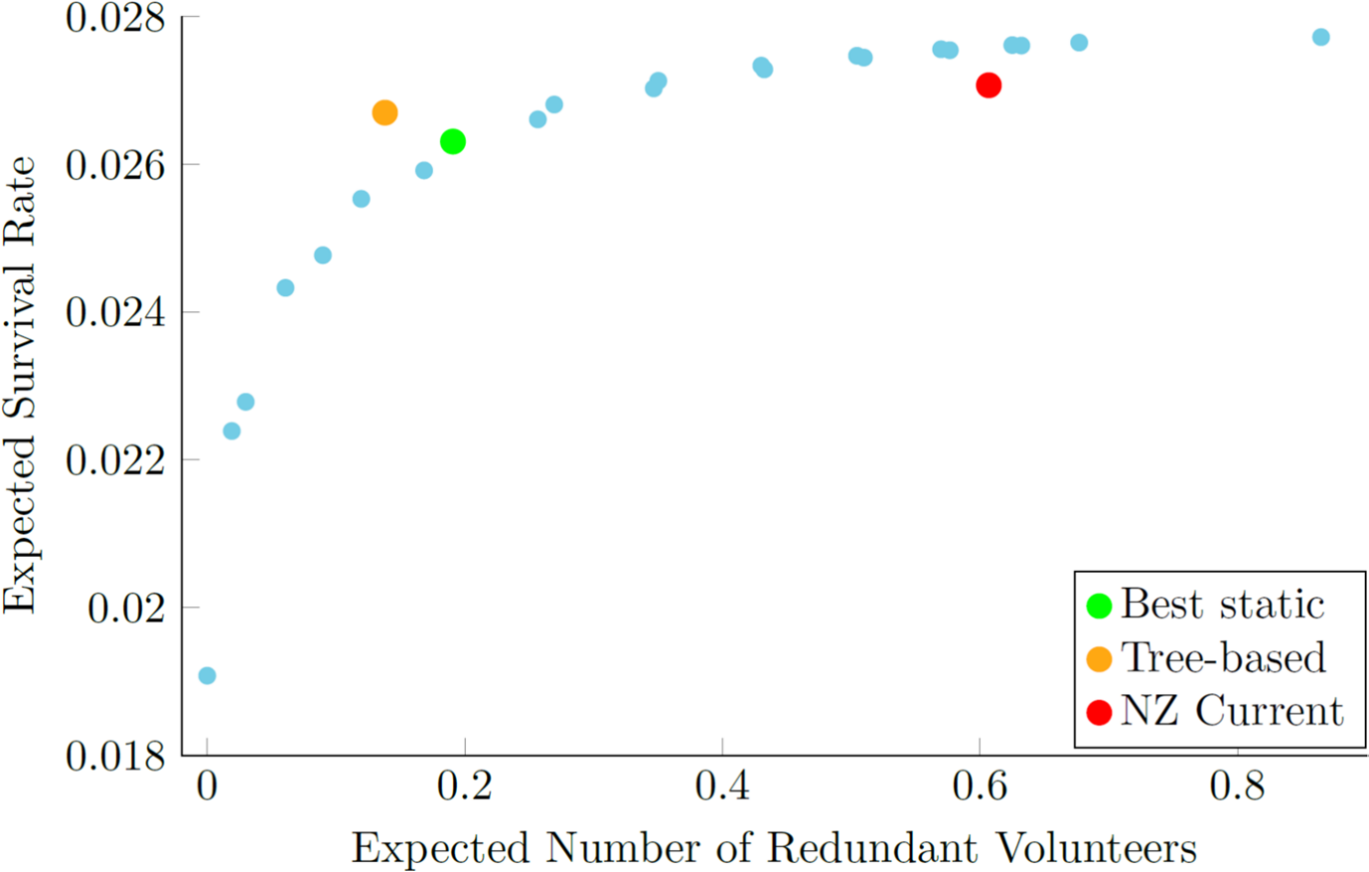
Trade-off between patient survival and number of redundant volunteers for static and adaptive tree-based strategies under a 14% acceptance rate and a 12-minute EMS response time. Each blue dot represents the performance of one static strategy. The orange dot indicates the performance of the tree-based strategy. The green dot indicates the performance of the best static strategy, which is *Send 4 at time 0*. The red dot indicates the performance of the current SJAS strategy

In all scenarios, we observed a 1-4% improvement in average performance across test cases by selecting an appropriate policy based on real-time information, even if the tree-based strategy occasionally misclassifies the best policy for certain instances. We say that the tree-based policy “misclassifies” an instance if it picks a policy that was not the best one seen in the simulation results for that instance. Figure 4 illustrates the trade-off between patient survival and redundant volunteers for both our adaptive and static strategies under a 14% acceptance rate and a 12-minute EMS response time. As we see, to attain higher survival rates, one must accept the “cost” of more redundant volunteers, because higher survival rates arise when policies alert more CFRs earlier, prior to the first acceptance, which inevitably leads to more redundant arrivals. The tree-based selection strategy lies outside the efficient frontier of static strategies, because it can tailor the response to each OHCA, in contrast to static strategies. The best static strategy for $\gamma = 0.01$ is *Send 4 at time 0*. For other values of $\gamma$ another static strategy can become optimal. Our tree-based policy, trained under gamma = 0.01, continues to outperform all static policies, for $\gamma$ ranging from 0.0022 to 0.0307, so is robust to the choice of $\gamma$. Appendix D explores what happens when we select different values of $\gamma$ from the outset, where the training of classification trees and the selection of the best static policy are both dependent on $\gamma$. The tree-based policies seen there expand the set of achievable survival rate-redundant volunteer pairs, as expected, with important gains in both dimensions.

For comparison, we also evaluated the policy in use by GoodSAM in Auckland. SJAS informed us that at the time of study, this was: alert volunteers in batches of 3, and keep repeating this in 30-second intervals until at least one person accepts. The current dispatch strategy practiced by SJAS lies within the efficient frontier of static strategies, indicating that it is suboptimal. Higher survival rates could have been achieved with the same number of redundant volunteers. It achieved similar survival rates to our tree-based selection strategy, but required far more redundant volunteers.

**Fig. 5:**
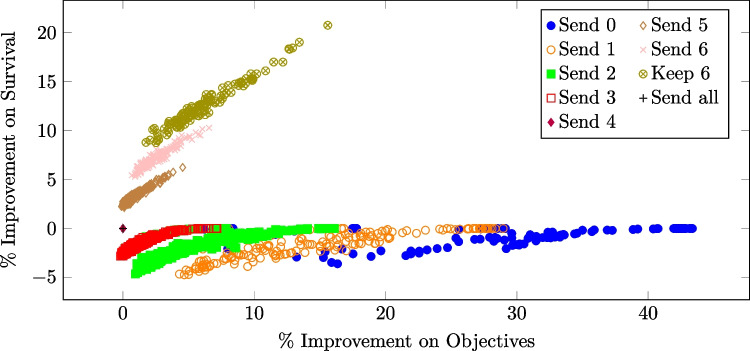
Percentage improvement in survival rate vs. the percentage improvement in the objective (2) of the tree-selected policy compared to the best static policy for 2000 test cases under a 14% acceptance and a 12-minute EMS response time. Each dot represents one test case and the color of the dot indicates, for that test case, the policy selected by the tree classifier

While the absolute difference in average performance over all test cases between the best static strategy and the selected tree-based strategy may be small, there can be significant improvements in individual instances. Figure 5 shows the percentage improvement in patient survival versus the percentage improvement in the objective of the tree-selected policy compared to the best static policy for each test case.

From Fig. 5, the tree-based policy almost universally improves on the best static policy, since almost all points lie to the right of 0 on the horizontal axis, but it can sometimes perform slightly worse than the best static policy, due to misclassification. The tree-based strategy is advantageous when there are some CFRs within the dispatch radius but even the closest CFR is still far from the patient. This is shown by the red and orange points on the right end of Fig. 5. In these instances, the tree-based strategy recognizes that a CFR response is unlikely to significantly improve patient survival, even with more alerts. When the potential improvement in survival is negligible, the strategy prioritizes conserving volunteer resources to reduce fatigue. Tree-based policies yield the most desirable results—improving both patient survival and the objective function value (2)—when there are a large number of CFRs within the dispatch radius who are relatively close to the patient. In these cases, the tree-based strategy selects a policy that sends out more alerts than the best static policy, leading to better outcomes that appear as points with positive improvements in survival in the plot.

### Impact of Volunteer Acceptance Rate

In the previous section, we worked with a 14% acceptance rate by directly bootstrapping CFR responses from the data. However, in areas with long-established CFR systems, the acceptance rate is likely to be higher. Consequently, we explore the impact of a higher volunteer acceptance rate on optimal volunteer dispatch strategies and their performance.

Let us assume an acceptance rate of 50%, which, according to a contact at GoodSAM, has been observed in the past in another city. When generating volunteer responses in our simulation, instead of directly bootstrapping from the data, we first sample a Bernoulli variable with a parameter of 0.5 to decide whether the response should be an accept or decline. Then, we repeatedly sample the CFR response from the data until the sampled CFR response matches the previously sampled Bernoulli outcome (accept or decline), and adopt the accompanying view delay from the data. This method boosts the acceptance rate from 14% to 50% while preserving the relationship between volunteer response and view delay.

Table 2 provides the accuracy of our tree-based strategy on the testing set and its average objective value, compared to the best static strategy under three different EMS response times and a 50% acceptance rate. Compared to Table 1, we can see that a higher acceptance rate leads to better performance for both the tree-based and best static strategies. The improvement over the best static strategy is around 3-5% in all three cases.

**Table 2:** Accuracy and average objectives reported by the tree-based selection strategy versus the average objective of the best static choice from our list under a 50% volunteer acceptance rate

EMS	Best static				Tree			
	Policy	Obj	Surv	Redundant	Obj	Surv	Redundant	Accuracy
6 min	Send 1 at time 0	0.1070	0.1101	0.3100	0.1100	0.1110	0.0966	0.991
9 min	Send 2 at time 0	0.0547	0.0595	0.4761	0.0574	0.0596	0.2218	0.958
12 min	Send 2 at time 0	0.0318	0.0352	0.3400	0.0329	0.0355	0.2577	0.939

Under the same EMS response time as before, with a higher CFR acceptance rate, the best static strategy sends out the same number or fewer alerts. This is because a higher acceptance rate could potentially result in a greater number of redundant volunteers. Although not shown in the table, *Keep n active until t* policies appear more frequently within the tree-based selection strategy as we increase the acceptance rate from 14% to 50%. With a higher acceptance rate, it is more beneficial to alert fewer CFRs initially to avoid volunteer fatigue. If the alerted CFR rejects, there is still a reasonable confidence that the next CFR will be available to ensure a fast response. The tree accuracy is higher with the higher acceptance rate, perhaps because there are fewer competitive policies and thus less opportunity to misclassify. Moreover, we see increasing tree accuracy as the EMS response time decreases, presumably for the same reason, as also seen in Table 1.

**Fig. 6:**
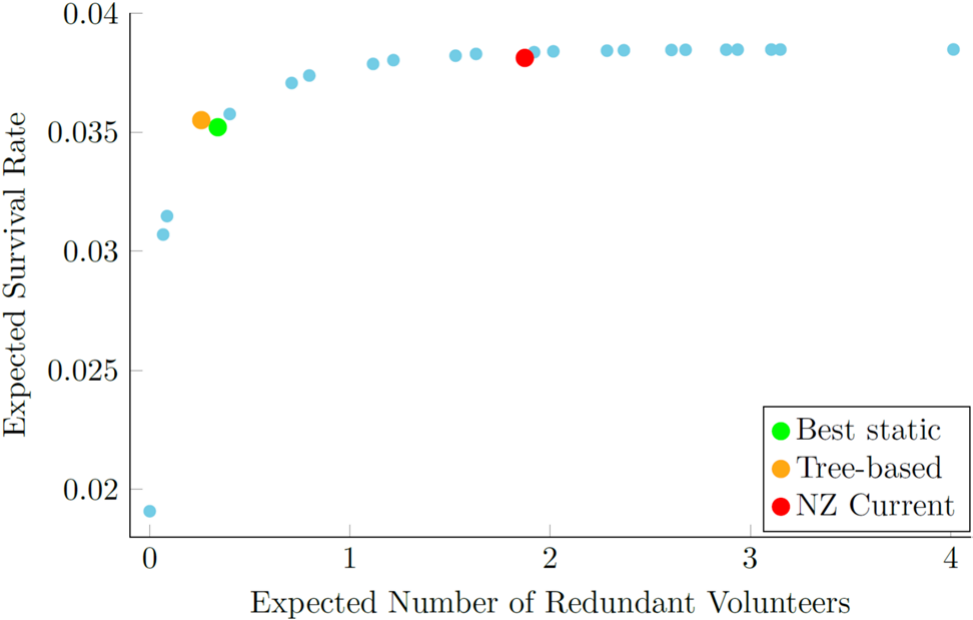
Trade-off between patient survival and number of redundant volunteers for static and adaptive tree-based strategies. Each blue dot represents the performance of one static strategy. The orange dot indicates the performance of the tree-based strategy. The green dot indicates the performance of the best static strategy, which is *Send 2 at time 0*. The red dot indicates the performance of the current SJAS strategy under a 50% acceptance rate and a 12-minute EMS response time

Figure 6 illustrates the trade-off between patient survival and redundant volunteers for both our adaptive and static strategies under a 12-minute EMS response time and a 50% acceptance rate. Similar to Fig. 4, the current SJAS strategy still lies within the efficient frontier under the higher volunteer acceptance rate, suggesting that it remains suboptimal. The tree-based selection strategy lies outside the efficient frontier of the static strategies. The tree-based selection strategy decreases the redundancy rate relative to the best static policy, which is *Send 2 at time 0*. It also yields a small improvement in patient survival, although the improvement is less significant than in Fig. 4 where the volunteer acceptance rate is lower. This is likely because, with such a high volunteer acceptance rate, the best static strategy already performs very well in terms of patient survival. As a result, the primary way to improve the overall objective is to reduce redundant volunteers without significantly increasing, or potentially even at the risk of compromising, patient survival.

**Fig. 7:**
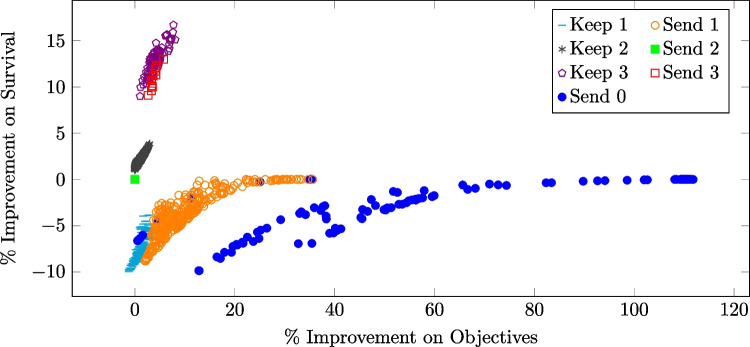
Percentage improvement in survival rate vs. the percentage improvement in the objective (2) of the tree-selected policy compared to the best static policy for 2000 test cases under a 50% acceptance and a 12-minute EMS response time. Each dot represents one test case and the color of the dot indicates, for that test case, the policy selected by the tree classifier

Figure 7 makes this observation more clear, showing the percentage improvement in patient survival versus the percentage improvement in the objective of the tree-selected policy compared to the best static policy for each test case under a 50% acceptance rate. The tree-based strategy improves upon the best static strategy in most cases by decreasing the number of redundant volunteers, at the cost of a decrease in patient survival. Only in a small number of cases, shown as the cluster of blue and olive points in the upper left corner of Fig. 7, does the tree-selected policy improve both survival and the overall objective. This typically occurs when there is a high CFR density within the dispatch radius. Compared to Fig. 5, Figure 7 more clearly demonstrates that the tree-based strategy is most advantageous in two scenarios: when there are a large number of CFRs who are relatively close to the patient as represented by points in the upper left corner, and when there are some CFRs within the dispatch radius but the closest CFR is far from the patient, as represented by most of the points below the horizontal axis, especially those to the right-hand end.

### MDP optimal policies vs. tree-selected policies

Next, we show that with a good pre-defined set of policies, the tree-based selection strategy can perform nearly “optimally." To align with the MDP setup, the tree-based selection strategy was trained on 10,000 instances with the same instance generation process as described in Section 5.3. However, given the computational time of MDP optimal policies, we restrict ourselves to instances with 6 or fewer volunteers that can reach the patient before ambulance arrival. We assumed a volunteer acceptance probability of 0.00105 and a rejection probability of 0.00221 at each 5s time step . When one computes the cumulative effect of these acceptance and rejection probabilities over the course of the 12 minute EMS response time, which consists of 144 time steps, we obtain overall probabilities of acceptance and rejection that match the empirical rates in the dataset.

**Fig. 8:**
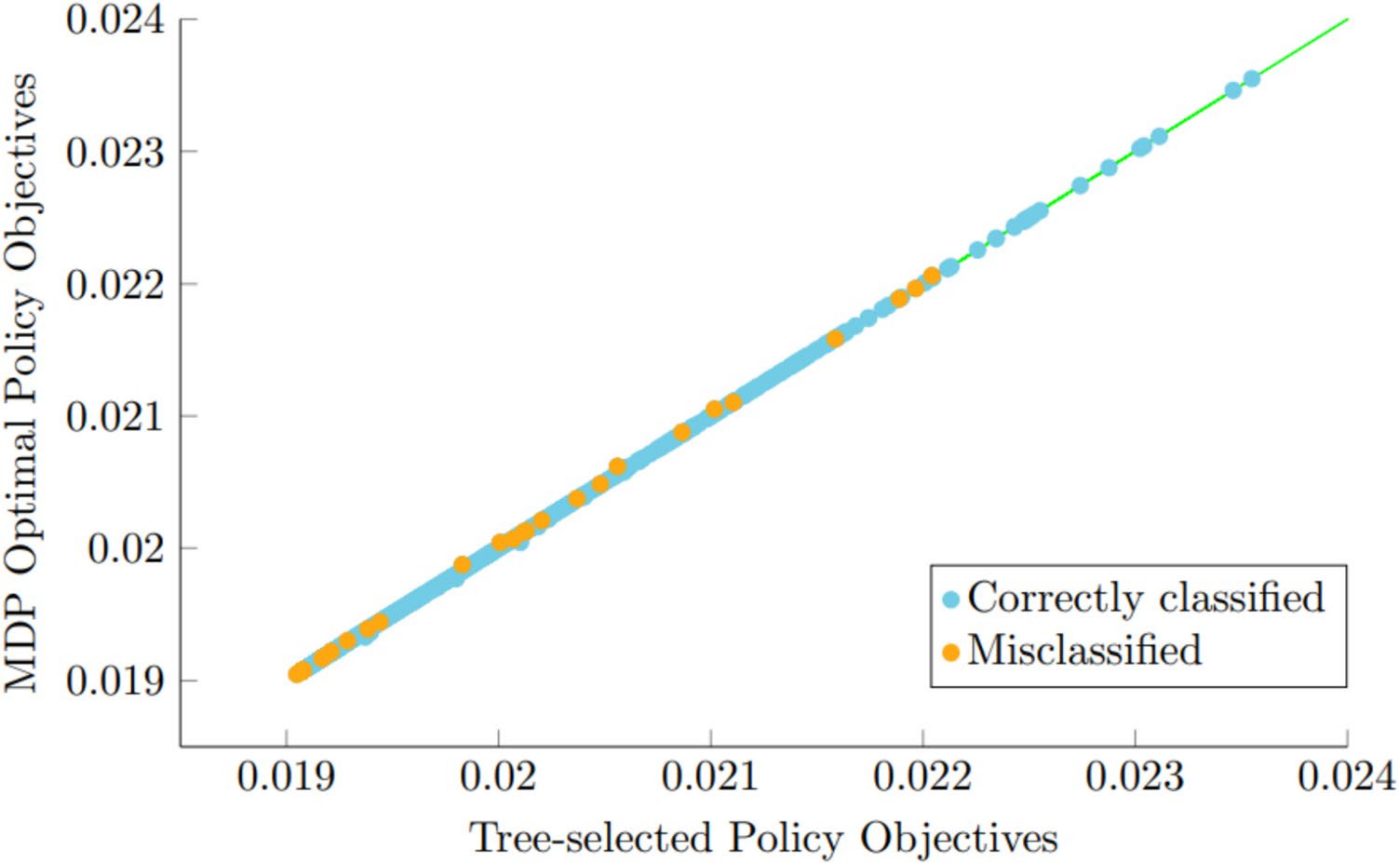
Performance of MDP optimal policies vs. performance of tree-selected policies on 1000 test cases under a 12-minute EMS response time and a 14% acceptance rate within 15 minutes of CFR system activation. The green line is the $y=x$ line. The orange dots represent instances where the tree-based strategy misclassifies the “best" policy among the pre-defined set. When misclassifications occur they are very small, as evidenced by the very small departures from the “$y=x$” line

Figure 8 compares the performance of MDP optimal policies versus tree-selected policies for 2000 test instances under a 12-minute EMS response time. In most instances, the tree-selected policy matches the performance of the MDP policy, which is optimal under this setting. Even when misclassification occurs, the tree-selected policy performs nearly optimally. The strong performance of our tree-based selection policy in this setting may be due to the fact that our pre-defined set of policies was constructed using characteristics observed from the MDP optimal policies. This suggests that with a well-chosen set of pre-defined policies that sufficiently covers the space of effective volunteer dispatching policies, the tree-based selection strategy can perform exceptionally well.

## Conclusion and discussion

In this paper, we introduced a discrete-event simulation model for evaluating CFR dispatch policies. We presented an MDP formulation as well as a data-driven method that uses simulation and a classification tree to learn the “optimal" CFR dispatch strategies. We demonstrated the performance of our proposed method through a comprehensive case study in Auckland in Section 5. While the results and insights for our tree-based strategy are specific to Auckland (Sections 6.1 and 6.2), the same selection strategy approach can be applied to other regions with data or with reasonable assumptions on incident rates and volunteer response distributions, as shown in Sections 6.3 and 6.4.

The MDP model presented in Section 3 determines which CFRs to alert and when, based on the estimated travel times of CFRs and assigned ambulances to the patient at the time of dispatch. Propositions 3.2 and 3.4 highlight key characteristics of MDP optimal policies: no additional alerts after an acceptance, and a threshold on the travel time of the closest unalerted volunteers determines whether an additional alert will be sent. Although computing the MDP optimal policies in real-time for each OHCA is unrealistic, these characteristics are useful for constructing the pre-defined policies for the selection strategy introduced in Section 4.

A selection strategy using a classification tree, or any classification method, can be trained using the simulation model presented in Section 4 to evaluate the performance of each policy for OHCA in the pre-defined set. Tables 1 and 2 show that shorter EMS response times result in fewer alerts being sent and simpler dispatch policies being optimal, given the shorter time for CFRs to respond. Figures 4 and 6 demonstrate how our tree-based adaptive selection strategy improves upon the fixed strategies currently used by CFR systems in practice.

An important observation from Section 6 is that the tree-selected strategy is not always desirable because it can misclassify, or it can choose to compromise patient survival for reduced volunteer fatigue. The selection strategy is most advantageous relative to the best static strategy in two extreme types of OHCA instance: when CFR response is extremely effective, leading to sending out many more alerts than the best static strategy, or when CFR response is unlikely to contribute much to patient survival, leading to refraining from sending alerts to reduce volunteer fatigue. Additionally, Section 6.3 highlights that volunteer acceptance rates heavily impact optimal dispatch policies and their performance. Higher acceptance rates lead to fewer alerts being sent but improved survival and performance overall compared to lower acceptance rates.

The selection strategy approach is highly adaptive to various extensions, beyond the assumptions made herein. For example, while we assumed a fixed volunteer travel speed, with sufficient data or reasonable assumptions about how volunteer travel speeds are distributed, we could evaluate the performance of each policy under random travel times. Additionally, both CFR system triage time and EMS response time significantly impact patient survival. Our simulation model can accommodate the evaluation of dispatch policies under both fixed and random triage and EMS response times. Our training for the selection strategy also allows for the inclusion of estimated EMS travel time as an additional feature to predict the appropriate policy for each incident.

There are many important directions for future research that could extend our selection strategy approach. First, we considered all volunteer arrivals after the first responder as redundant. However, high-quality CPR can be tiring, and having more than one volunteer to alternate can be beneficial. How would the optimal dispatch policies change if we start counting redundant volunteers after the first *k* arrivals? The tree-based selection strategy could be adapted to this setting by modifying how we evaluate redundant volunteers in the simulation model.

In our objective we quantified volunteer fatigue as redundant arrivals, but what really captures volunteer disengagement? More specifically, can one quantify how this disengagement decreases survival for future patients? We are not aware of any work on this topic and encourage future research that could subsequently inform the design of dispatch policies.

We trained our classification trees to maximize the probability of selecting the best policy, but one may experiment with different ways to capture the loss in classification. For example, one might want to select a policy that is no more than $\delta$ from the best policy [13], which is equivalent to maximizing the probability of finding a *good* solution. The latter is of interest but is not embedded in standard CART packages and therefore would warrant new methodological research.

In the context of OHCA, the availability of an AED significantly impacts survival. One may wish to extend our work to more complex policies that include pickup of AEDs by some volunteers. What survival benefits could be expected if we dispatch some CFRs to grab a publicly available AED on the way to the patient? What would be considered a “good" set of policies to start with for our selection strategy in this setting? Although the GoodSAM New Zealand app does not currently offer such directions, it would be a useful addition in countries where a good-quality AED registry exists. We provide an initial exploration of AED policies in the appendix to demonstrate feasibility, but leave a comprehensive exploration of policies to future work.

Additionally, off-duty healthcare professionals and firefighters are potentially more willing to accept an alert compared to the general public with only CPR training. How would dispatch policies change if we consider different classes of CFRs with varying acceptance probabilities and abilities to help? How do we balance the proximity of the CFR to the patient with their willingness and level of proficiency? These questions would require a model with multiple classes of CFRs, which could also be incorporated into our selection strategy approach by adding volunteer classes as predictors.

A recent podcast [24] reports an overall 8% survival rate for OHCA patients in the USA, but in specific locations like casinos, the survival rate can reach as high as 54% due to a very fast response by well-trained employees. Hence, in some very special locations, there is no need to alert additional CFRs. How valuable would it be to consider the location of the OHCA as a factor in deciding the appropriate dispatch policy to use? Exploring these questions could lead to more nuanced and effective dispatch strategies, ultimately improving patient outcomes and volunteer efficiency.

## Figures and Tables

**Fig. 9:**
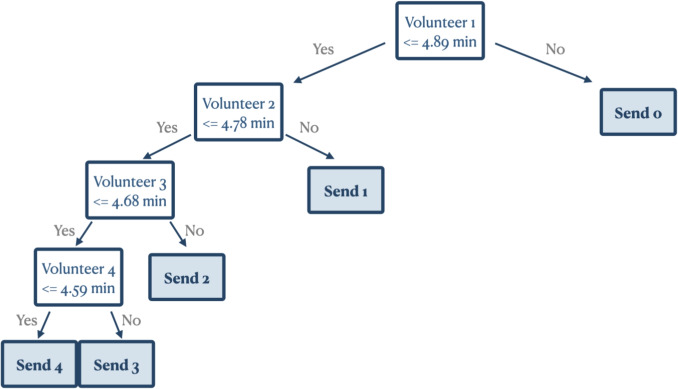
Tree-based selection strategy for instances in Auckland with 14% acceptance rate and 6-minute EMS response time measured from call arrival. Volunteers are numbered by how long they would take to reach the OHCA, with Volunteer 1 being the closest. So, for example, if there are 5 volunteers with response times (3, 5, 8, 9, 10) minutes, respectively, then the policy chosen is “Send 1.” And if there are 5 volunteers all with response times under 4 minutes, then the policy chosen is “Send 4.”

**Fig. 10:**
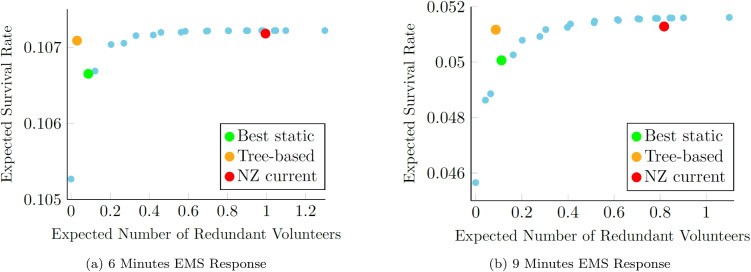
Trade-off between patient survival and number of redundant volunteers for static and adaptive tree-based strategies under a 14% acceptance rate. Each blue dot represents the performance of one static strategy. The orange dot indicates the performance of the tree-based strategy. The green dot indicates the performance of the best static strategy, which is *Send 1 at time 0* under a 6-minute EMS response time (left) and *Send 2 at time 0* under a 9-minute EMS response time (right). The red dot indicates the performance of the current SJAS strategy

**Fig. 11:**
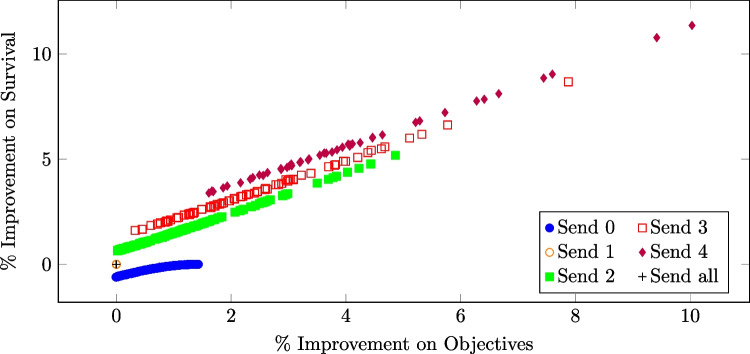
Percentage improvement in survival rate vs. the percentage improvement in the objective (2) of the tree-selected policy compared to the best static policy for 2000 test cases under a 14% acceptance and a 6-minute EMS response time

**Fig. 12:**
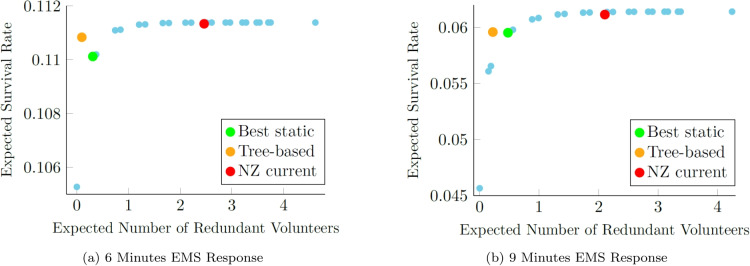
Trade-off between patient survival and number of redundant volunteers for static and adaptive tree-based strategies under a 50% acceptance rate. Each blue dot represents the performance of one static strategy. The orange dot indicates the performance of the selected tree-based strategy. The green dot indicates the performance of the best static strategy, which is *Send 1 at time 0* under a 6-minute EMS response time (left) and *Send 2 at time 0* under a 9-minute EMS response time (right). The red dot indicates the performance of the current SJAS strategy

**Fig. 13:**
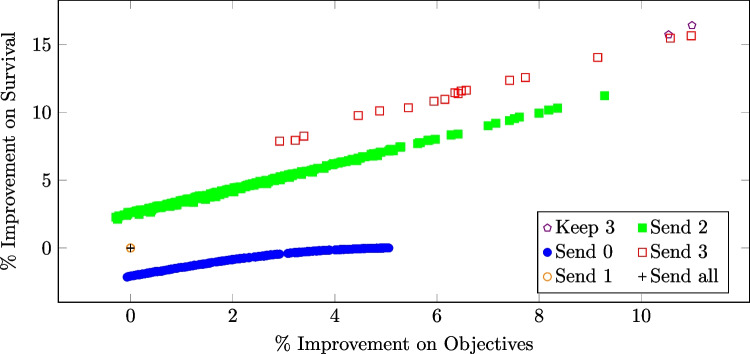
Percentage improvement in survival rate vs. the percentage improvement in the objective (2) of the tree-selected policy compared to the best static policy for 2000 test cases under a 50% acceptance and 6-minute EMS response time

**Fig. 14:**
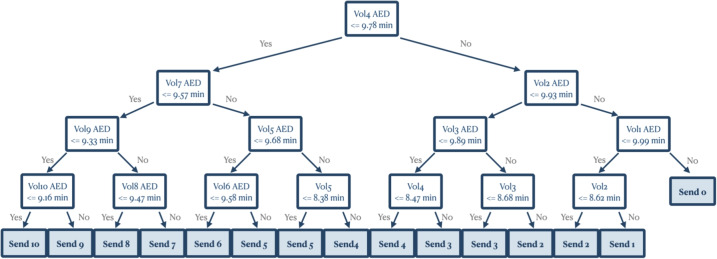
Tree-based selection strategy for instances in Auckland with 14% acceptance rate and 12-minute EMS response time measured from call arrival. Volunteers are numbered by how long they would take to reach the OHCA, with Volunteer 1 being the closest. So, for example, if there are 5 volunteers with direct response times (3,5,8,9,10) minutes and AED response times (5, 7, 9, 10, 11) minutes, respectively, then the policy chosen is “Send 3.” And if there are 5 volunteers all with AED response times under 8 minutes, then the policy chosen is “Send 5.”

**Fig. 15:**
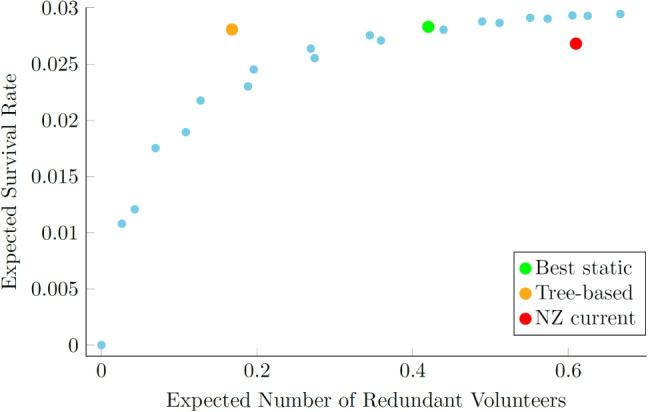
Trade-off between patient survival and number of redundant volunteers for static and adaptive tree-based strategies with AEDs incorporated, under a 14% acceptance rate and a 12-minute EMS response time. Each blue dot represents the performance of one static strategy. The orange dot indicates the performance of the tree-based strategy. The green dot indicates the performance of the best static strategy, which is *Send 7 at time 0*. The red dot indicates the performance of the current SJAS strategy

**Fig. 16:**
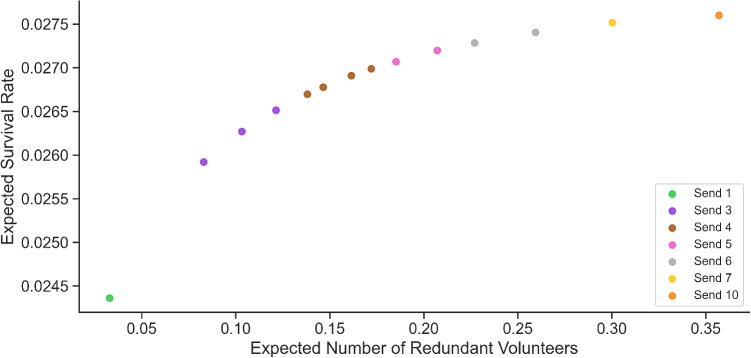
Trade-off between patient survival and number of redundant volunteers for tree-based selection strategy under a 14% acceptance rate and 12-minute EMS response. Each dot represents the performance of a tree-based selection strategy with $\gamma$ value, when viewing the dots from left to right, of (0.05,0.02,0.015,0.012,0.01,0.009,0.008,0.007,0.006,0.005,0.004,0.003,0.002,0.001). The color of the dot indicates the best static strategy corresponding to that $\gamma$

## Data Availability

All data used in this paper will be made available upon request, except for the empirical data from the GoodSAM community first response system, which we are not able to share unless the interested reader seeks approval from Hato Hone St John Ambulance Service.

## Appendix A. Proofs for the markov decision process formulation

In this appendix, we provide the proofs for the propositions presented in Section 3, which describe important characteristics of the MDP optimal policies. We then present another interesting property of the MDP formulation when volunteer fatigue is defined differently.

### Proposition 3.1

The Pareto-optimal frontier is concave with respect to the expected volunteer fatigue for any fixed OHCA.

### Proof

Consider any single OHCA. Let $\pi _1, \pi _2$ be two policies with $(g_{\pi _1}, f_{\pi _1}), (g_{\pi _2}, f_{\pi _2})$ being the expected volunteer fatigue and expected survival probability for the two policies $\pi _1, \pi _2$ respectively. For any $\lambda \in [0,1]$, consider the randomized policy $\pi _\lambda$ that dispatches under policy $\pi _1$ with probability $\lambda$ and dispatches under policy $\pi _2$ with probability $1-\lambda$, independently of all else. Under policy $\pi _\lambda$, the expected volunteer fatigue is $\lambda g_{\pi _1} + (1-\lambda ) g_{\pi _2}$, and the expected survival probability is $\lambda f_{\pi _1} + (1-\lambda ) f_{\pi _2}$. Thus, the set $G = \{(g, f): (g, f) \text { is attained by a policy}\}$ is convex. The result follows immediately. $\square$

### Proposition 3.2

Once someone has accepted, the optimal policy $\pi *$ will not send out any additional alerts.

### Proof

Consider a state $s_t = (t, \textbf{v}_t, r_t)$, where *m* volunteers have been alerted, and at least one volunteer has accepted the alert. Let $\pi$ be a policy that issues an additional alert in state $s_t$. Contrast this with a policy $\pi '$ that sends no further alerts in state $s_t$ from time *t* onwards. The values of $s_t$ under policies $\pi$ and $\pi '$, using (2), are

$$\begin{aligned} V^{\pi }(s_t)&= f_\pi (s_t) - \gamma g_\pi (s_t), \text { and}\\ V^{\pi '}(s_t)&= f_{\pi '}(s_t) - \gamma g_{\pi '} (s_t), \end{aligned}$$

where $f_{\pi }(s_t)$ and $f_{\pi '}(s_t)$ represent the expected survival probability, and $g_{\pi }(s_t)$ and $g_{\pi '}(s_t)$ represent the expected volunteer fatigue given the state $s_t$ following policies $\pi ,\pi '$ respectively.

The expected survival under policies $\pi$ and $\pi '$ remains the same because any additional volunteer alerted by $\pi$ is further away than the responding volunteer. However, $\pi$ sends more alerts, resulting in a higher number of expected redundant volunteers ($g_{\pi }(s_t) \ge g_{\pi '}(s_t)$). As a result, $V^{\pi }(s_t) \le V^{\pi '}(s_t)$, indicating that $\pi$ is never optimal. $\square$

Recall, from Section 3, that *s*(*d*, *t*) and *r*(*d*, *t*) represent the expected increase in survival probability and the probability of arriving after the ambulance when considering a single volunteer located *d* time steps from the patient who is alerted at time *t*. In addition, recall the threshold *d*(*t*):

### Definition 3.3

Let d(t) be the minimum number of travel time steps such that any volunteers located beyond this threshold have an expected benefit that is less than the expected penalty, i.e.,

7 $$\begin{aligned} d(t) = \text {min}\{d \epsilon \{0, 1, . . . , T\}: s(d, t) < yr(d, t)\}. \end{aligned}$$

We observe the following monotonicity properties.

### Lemma Appendix A.1

For a fixed time step *t*, as *d* increases,

(i) *s*(*d*, *t*) decreases monotonically, and
(ii) *r*(*d*, *t*) increases monotonically.

### Lemma Appendix A.2

The threshold *d*(*t*) is decreasing in *t*.

The proofs of the above lemmas are intuitive and straightforward. A volunteer located further away or alerted later in time has less time to respond and arrives later, which causes a smaller contribution to survival and a higher probability of arriving after the ambulance.

### Proposition 3.4

Suppose the i-th volunteer, located Di time steps away from the patient, is the closest unalerted volunteer. The optimal policy $\pi *$ will not alert this volunteer at time t if $Di \ge d(t)$.

### Proof

Let $s=(t, \textbf{v}, r)$ be a non-terminal state such that the *i*-th volunteer is the closest unalerted volunteer located $D_i$ time steps away from the patient. If someone has already accepted the alert, then by Proposition 3.2, we are done. Now consider the case where none of the alerted volunteers have accepted the alert. Given $D_i \ge d(t)$, by Lemma Appendix A.1 we know $s(D_i, t) < \gamma r(D_i, t)$.

We consider induction on the remaining travel time steps of the fastest responder *r*. The induction begins at $r=3$ because, for $r\le 2$, even if the closest non-alerted volunteer has a travel time of 0 and accepts the alert immediately, they can only arrive at the same time as the ambulance. (In the MDP setup, it takes one time step to reflect any change in the volunteer’s status.) In such cases, a newly alerted volunteer does not improve survival outcomes and becomes redundant immediately.

When $r=3$, the values of state *s* under different actions are

$$\begin{aligned} Q(s, a_t=0)&= \sum _{s'} p(s' | s, 0) \left[ h(s, 0, s') + V^*(s') \right] , \text { and}\\ Q(s, a_t=1)&= \sum _{s''} p(s'' | s, 1) \left[ h(s, 1, s'') + V^*(s'') \right] . \end{aligned}$$

Consider the volunteer status vectors $\mathbf {v'}$ and $\mathbf {v''}$ from $\textbf{v}$ following the action $a_t=0$ and $a_t=1$ respectively. The update to the volunteer status vector only depends on the $i-1$ previously alerted volunteers in $\textbf{v}$, as $a_t$ only determines whether the *i*-th volunteer is alerted or not. Therefore, there exists a one-to-one correspondence between $\mathbf {v'}$ and $\mathbf {v''}$, and thus a one-to-one correspondence between $s' = (t+1, \mathbf {v'}, r_{t+1})$ and $s'' = (t+1, \mathbf {v''}, r_{t+1})$. Note $s'$ and $s''$ share the same $r_{t+1} \le 2$ because either a previously alerted volunteer has just accepted the alert and shortened the fastest response time or $r_t=2$.

If $r_{t+1}=0$, then $s'$ and $s''$ become terminal states. In this case, $h(s,0, s') \ge h(s, 1, s'')$ because $s'$ and $s''$ share the same survival probability but $s''$ has an additional alerted volunteer, which increases the expected number of redundant volunteers.

If $s'$ and $s''$ are non-terminal, we know that $\pi ^*(s') = 0$ and $\pi ^*(s'')=0$, as discussed above, since $r_{t+1}\le 2$. Then, from (4), we have

$$ V^*(s'') - V^*(s') = \tilde{s} -\gamma \tilde{r}, $$

where $\tilde{s}$ is the marginal benefit of alerting the *i*-th volunteer, and $\tilde{r}$ is the probability that the *i*-th volunteer becomes redundant if alerted at *t*. We can see that $\tilde{s} \le s(D_i, t)$ because the benefit of alerting the *i*-th volunteer is realized only if they arrive before both the ambulance and all other alerted volunteers. Similarly, we have $\tilde{r} \ge r(D_i, t)$ because the *i*-th volunteer can become redundant not only by arriving after the ambulance but also by arriving after or at the same time as other alerted volunteers. Hence, by our given assumption, we have

$$ V^*(s'') - V^*(s') = \tilde{s} -\gamma \tilde{r} \le s(D_i, t) - \gamma r(D_i, t) < 0. $$

As a result, we can conclude that $V^*= Q(s, a_t=0) \ge Q(s, a_t=1)$ and thus $\pi ^*(s)= 0$.

Now assume the statement holds for $3, \dots , r-1$. Consider all possible next states $s' = (t+1, \mathbf {v'}, r_{t+1})$ and $s'' = (t+1, \mathbf {v''}, r_{t+1})$ resulting from the action $a_t=0$ and $a_t=1$ respectively.

By Lemma Appendix A.2, we have $$D_{i+1} \ge D_i \ge d(t) \ge d(t+1)$$. Therefore, it follows from the induction hypothesis that $$\pi ^*(s')=0$$ and $$\pi ^*(s'') = 0$$. Using a similar argument as in the base case, we know for all possible pairs of $$s'$$ and $$s''$$, either $$h(s,0,s') \ge h(s,1,s'')$$ (when $$s'$$ and $$s''$$ are terminal) or $$V^*(s') > V^*(s'') $$. We can then conclude that $$\pi ^*(s)=0$$.

A straightforward inductive argument completes the proof. $\square$

### Appendix A.1 Properties under a different definition of volunteer fatigue

So far, we have defined volunteer fatigue as proportional to the number of redundant volunteers on-scene. However, multiple factors could contribute to volunteer fatigue. Another possible factor is when volunteers receive too many alerts, leading to annoyance and disengagement. Therefore, penalizing the number of alerts sent can also be a way of defining volunteer fatigue.

Proposition Appendix A.3 indicates that there are diminishing returns when the status of volunteers remains unchanged over time, as this implies volunteers arrive later, which in turn results in a lower survival probability.

#### Proposition Appendix A.3

If volunteer fatigue is defined as proportional to the number of alerts sent, then when volunteer status remains the same, as time goes on, the value of states decreases, i.e. for any non-terminal states $s_t = (t, \mathbf {v_t}, r_t)$ and $s_{t+1} = (t+1, \mathbf {v_{t+1}}, r_{t+1}=r_t-1)$, we have $V^*(s_t) \ge V^*(s_{t+1})$.

Before we prove Proposition Appendix A.3, we first present the following lemma, which shows that the diminishing return holds even when someone just accepts the alert and the remaining fastest responder travel time becomes the same.

#### Lemma Appendix A.4

Consider a volunteer status vector $\textbf{v}$ such that at least one of the alerted volunteers has accepted the alert. For any non-terminal states $s_1=(t,\textbf{v},r)$ and $s_2 = (t+1, \textbf{v}, r)$, we have $V^*(s_1)\ge V^*(s_2)$.

#### Proof

We consider induction on the remaining travel time steps of the fastest responder *r*. When $r=1$, all possible next states of $s_1$ and $s_2$ are terminal. By Proposition 3.2, no alerts will be sent at states $s_1$ and $s_2$ ($\pi ^*(s_1)=\pi ^*(s_2)=0$). Let *m* be the total number of volunteers alerted in $\textbf{v}$. Then the values of $s_1$ and $s_2$ under the optimal policy $\pi ^*$, using (4), are

$$\begin{aligned} V^{*}(s_1)&= \phi (t_{CPR,1}, t_{EMS}) - \gamma m, \text { and}\\ V^{*}(s_2)&= \phi (t_{CPR,2}, t_{EMS}) - \gamma m, \end{aligned}$$

where $\phi (t_{CPR,1}, t_{EMS})$ and $\phi (t_{CPR,2}, t_{EMS})$ gives the expected survival rate of $s_1, s_2$ using (1), where $t_{EMS}$ is fixed for both states, and $t_{CPR,1}$ and $t_{CPR,2}$ are the minimum of the sums of $t+1$ and $t+2$ with the witness delay and $t_{EMS}$, respectively. $\pi ^*$ gives a higher expected survival rate for $s_1$ following (1), because $s_1$ is one time step prior to $s_2$ ($t_{CPR,1} \le t_{CPR,2}$) and thus has a higher probability of reaching the patient faster with the same first responder travel time. As a result, $V^*(s_1)\ge V^*(s_2)$.

Now assume Lemma Appendix A.4 holds for $1, \dots , r-1$. Similarly, we know from Proposition 3.2 that $\pi ^*(s_1)=\pi ^*(s_2)=0$. The values of $s_1$ and $s_2$ under the optimal policy $\pi ^*$, using (4), are

$$\begin{aligned} V^{*}(s_1)&=\sum _{s_1'} p(s_1' | s_1, 0) \left[ h(s_1, 0, s_1') + V^*(s_1') \right] , \text { and}\\ V^{*}(s_2)&= \sum _{s_2'} p(s_2' | s_2, 0) \left[ h(s_2, 0, s_2') + V^*(s_2') \right] . \end{aligned}$$

Consider all possible evolutions $\mathbf {v'}$ from the volunteer status vector $\textbf{v}$, given that there is no alert sent out at states $s_1$ and $s_2$, there is a one-to-one correspondence between $s_1'$ and $s_2'$ under the same volunteer status vector $\mathbf {v'}$. There are two scenarios to consider:

1. *No new volunteer acceptance that improves upon the fastest responder*: This scenario gives us $s_1' = (t+1, \mathbf {v'}, r-1)$ and $s_2' = (t+2, \mathbf {v'}, r-1)$.
2. *New acceptance improves upon the fastest responder*: Without loss of generality, assume it is volunteer *n* with $d_n < r-1$, which gives us $s_1' = (t+1, \mathbf {v'}, d_n)$ and $s_2' = (t+2, \mathbf {v'}, d_n)$.

In both scenarios, $s_1'$ and $s_2'$ are non-terminal, so no reward is accrued ($h(s_1, 0, s_1')=h(s_2, 0, s_2')=0$) according to (2). Since $V^*(s_1')\ge V^*(s_2')$ follows immediately from the induction hypothesis, we have $V^*(s_1)\ge V^*(s_2)$. A straightforward inductive argument completes the proof. $\square$

#### Proof of Proposition Appendix A.3

Similarly, we consider induction on the remaining travel time steps of the fastest responder $r_t$ in state $s_t$. We start with $r_t=2$ to ensure $s_{t+1}$ is non-terminal with $r_{t+1} = 1$. The optimal policy $\pi ^*$ does not send any alerts when $r=1$, because even if the alerted volunteer responds immediately, they will not improve upon the fastest responder who has arrived by the time of the response.

All possible next states of $s_{t+1}$ are terminal states. Let *m* be the total number of volunteers alerted in $\textbf{v}$. The value of $s_{t+1}=(t+1, \textbf{v}, 1)$, using (4), is given by

$$ V^*(s_{t+1}) = \sum _{s'} p(s' | s_{t+1}, 0) \left[ h(s_{t+1}, 0, s') + V^*(s') \right] = \phi (t_{CPR},t_{EMS}) - \gamma m, $$

where $t_{CPR}$ is the minimum of the sum of $t+2$ with the witness delay and $t_{EMS}$.

Now consider the value of state $s_t$ if no alert is sent at $s_t$ ($a_t=0$),

$$ Q(s_t, a_t=0) = \sum _{s'} p(s' | s_{t}, 0) \left[ h(s_{t}, 0, s') + V^*(s') \right] . $$

We know $Q(s_t, a_t=0) \le V^*(s_t)$ following the definition of the optimal policy in (5). If $\textbf{v}$ has no outstanding alerts, i.e., alerts with no response, then the only possible next state of $s_t$ is $s_{t+1}$ and $Q(s_t,a_t=0) = V^*(s_{t+1})$. Even if there exist outstanding alerts, they will not improve upon the response time as we discussed above, i.e., $s' = (t+1, \mathbf {v'}, 1)$. Then $Q(s_t,a_t=0) = V^*(s_{t+1})$ because all possible evolutions $\mathbf {v'}$ of the volunteer status vector $\textbf{v}$ has *m* alerts sent out under the optimal policy $\pi ^*$ and all possible next states $s'$ and $s_{t+1}$ have the same volunteer response time. As a result, $V^*(s_t) \ge Q(s_t,a_t=0) = V^*(s_{t+1})$.

Now assume Proposition Appendix A.3 holds for $2, \dots , r_t-1$. Denote the action taken by the optimal policy $\pi ^*$ at state $s_{t+1}$ as $a^*_{t+1}=\pi ^*(s_{t+1})$. The value of $s_t$ following action $a^*_{t+1}$ is no larger than the value of $s_t$ under the optimal policy $\pi ^*$, i.e., $Q(s_t, a_t=a^*_{t+1}) \le V^*(s_t)$ following (4) and (5):

$$\begin{aligned} Q(s_t, a_t=a^*_{t+1})&=\sum _{s_t'} p(s_t' | s_t, a^*_{t+1}) \left[ h(s_t, a^*_{t+1}, s_t') + V^*(s_t') \right] , \text { and}\\ V^{*}(s_{t+1})&= \sum _{s_{t+1}'} p(s_{t+1}' | s_{t+1}, a^*_{t+1}) \left[ h(s_{t+1}, a^*_{t+1}, s_{t+1}') + V^*(s_{t+1}') \right] . \end{aligned}$$

Consider all volunteer evolutions $\mathbf {v'}$ from $\textbf{v}$ following the action the $a^*_{t+1}$. This only depends on the *m* alerted volunteers in $\textbf{v}$ as $a^*_{t+1}$ only decides whether the $m+1$-th closest volunteer is alerted or not. Similarly, there is a one-to-one correspondence between $s_t'$ and $s_{t+1}'$, the possible next states of $s_t$ and $s_{t+1}$, respectively, if they have the same volunteer status vector $\mathbf {v'}$. There are two scenarios to consider:

1. *No new volunteer acceptance that improves upon the fastest responder*: This scenario gives us $s_t' = (t+1, \mathbf {v'}, r_t-1)$ and $s_{t+1}' = (t+2, \mathbf {v'}, r_t-2)$.
2. *New acceptance improves upon the fastest responder*: Without loss of generality, assume it is volunteer *n* with $d_n \le r_t-2$, which gives us $s_t' = (t+1, \mathbf {v'}, d_n)$ and $s_{t+1}' = (t+2, \mathbf {v'}, d_n)$.

Under both scenarios, $s_t'$ and $s_{t+1}'$ are non-terminal, so no reward is accrued ($h(s_t, a^*_{t+1}, s_t')=h(s_{t+1}, a^*_{t+1}, s_{t+1}')=0$) according to (2). $V^*(s_t')\ge V^*(s_{t+1}')$ follows immediately from the induction hypothesis and Lemma Appendix A.4, respectively. Then, we have $V^*(s_t) \ge Q(s_t, a_t = a^*_{t+1}) \ge V^*(s_{t+1})$. A straightforward inductive argument completes the proof. $\square$

#### Proposition Appendix A.5

If all alerted volunteers have rejected alerts by time *t* and, under the optimal policy $\pi ^*$, no new alert is sent at time *t*, then no alerts will be sent out afterward.

#### Proof

Let $\textbf{v}$ be a volunteer status vector such that all *m* alerted volunteers have rejected the alert. Consider $s_t = (t,\textbf{v},r)$, a non-terminal state where the optimal policy does not send out any additional alert in state $s_t$, i.e. $\pi ^*(s_t)=0$.

We consider the potential next states for different actions. If $a_t=0$, i.e., no alert is sent, then $s_t$ transitions to state $s_{t+1} = (t+1, \textbf{v}, r-1)$ with probability 1. If, instead, $a_t=1$, i.e., the next closest volunteer is alerted, then, letting $\textbf{v}'$ be the volunteer status vector updated to reflect the newly alerted volunteer, the system transitions to state $s'_{t+1} = (t+1, \textbf{v}', r-1)$ with probability 1.

Given that the optimal policy does not send out any alerts in state $s_t$, we know

A.1 $$\begin{aligned} V^*(s_t)=V^*(s_{t+1}) > V^*(s'_{t+1}). \end{aligned}$$

Now suppose the optimal policy $\pi ^*$ issues an additional alert in the state $s_{t+1}$, i.e. $\pi ^*(s_{t+1})=1$. Then, the only possible next state of $s_{t+1}$ is $s'_{t+2} = (t+2, \textbf{v}', r-2)$, and

$$ V^*(s_{t+1}) = V^*(s'_{t+2}) \le V^*(s'_{t+1}). $$

The inequality follows directly from Proposition Appendix A.3. This contradicts Equation A.1 above. Thus it is not optimal to issue a new alert at time $t+1$. A straightforward inductive argument completes the proof. $hfill\square$

## Appendix B. Numerical results for shorter EMS response times

In this appendix, we present the performance of our proposed tree-based selection strategy under shorter EMS response times and compare it with the results shown in Section 6. First, we provide an example of the tree-based selection strategy under a 6-minute EMS response in Appendix B.1. We then illustrate the performance of the tree-based strategy under shorter EMS response times compared to static strategies in Appendi B.2. Finally, we conclude this appendix with an analysis of the impact of a high acceptance rate on dispatch policies in Appendix B.3.

### Appendix B.1. Tree-based strategy with shorter EMS response times

Figure 9 shows an example of the tree-based selection strategy trained on generated instances in Auckland with a 6-minute EMS response time and a 14% acceptance rate. This is a much shorter EMS response time, which SJAS aims to meet for 50% of incidents. Similar to Fig. 3, the resulting tree-based strategy is highly interpretable. As mentioned in Section sec:adaptive, Figure 9 is somewhat simpler than the tree for a 12-minute EMS response time, with fewer varieties of policies selected. *Keep n active until time t* policies are never selected because there is no time to wait for CFR response.

When deciding which policy to use, Figure 9 focuses more on the closer CFRs compared to Fig. 3 because, with such a short response time after the system triage, only the closer CFRs could potentially contribute to patient survival. Additionally, CFR system triage delay now becomes more significant. The tree in Fig. 3 never splits on triage delay because CFRs still have a sufficient amount of time to respond, even after accounting for the triage delay. However, with a 6-minute EMS response time, triage delay can significantly impact the amount of time CFRs have left to respond, even though Fig. 9 does not split on the triage time.

### Appendix B.2. Adaptive tree-based strategies vs. static strategies

Next, we compare the performance of our adaptive tree-based strategy with static strategies under both a 6-minute and a 9-minute EMS response time. We also present an instance-wise plot showing the percentage improvement of the tree-selected policy over the best static policy under a 6-minute EMS response time. We compare these results to those shown in Section 6.2.

Figure 10a and 10b illustrate the trade-off between patient survival and redundant volunteers for both our adaptive and static strategies under a 14% acceptance rate, with 6-minute and 9-minute EMS response times, respectively. Under both EMS response times, the tree-based selection strategy lies far outside the efficient frontier of the static strategy, improving patient survival rates and reducing redundant volunteers, while the SJAS current strategy lies within the frontier.

As shown in both Figs. 10a and  10b, the tree-based selection strategy also achieves similar survival rates to the current SJAS strategy, but with many fewer redundant volunteers. Additionally, the tree-based selection strategy can achieve a higher survival rate than the best static strategy, which is *Send 1 at time 0* under a 6-minute EMS response time and *Send 2 at time 0* under a 9-minute EMS response time, while having a smaller number of redundant volunteers.

Figure 11 makes this observation more clear, showing the percentage improvement in patient survival versus the percentage improvement in the objective of the tree-selected policy compared to the best static policy for each test case under a 14% acceptance rate and a 6-minute EMS response time. Compared to Fig. 5, the tree-based strategy under a 6-minute EMS response time improves upon the best static strategy in most cases by increasing both the overall objective and patient survival through sending out more alerts. Only in a small number of cases, shown as the cluster of red points in the lower left corner of Fig. 11, does the tree-selected policy choose to decrease the number of redundant volunteers. Given that the best static policy is *Send 1 at time 0*, this typically occurs when the time left for CFRs to respond is too short or when even the closest CFR is too far away. In these cases, it becomes optimal to rely solely on the ambulance response without any volunteer contribution. Compared to Fig. 5, Figure 11 shows that the tree-based strategy is most advantageous when there is a high CFR density within the dispatch radius. Since the best static policy is already quite conservative in sending alerts, there is limited potential for improving overall objectives by reducing the number of alerts.

### Appendix B.3 Impact of high volunteer acceptance rate

In the previous section, we assumed a 14% acceptance rate by directly bootstrapping CFR responses from the data. We now explore the impact of a higher volunteer acceptance rate on optimal volunteer dispatch strategies and their performance under shorter EMS response times. We assume a 50% acceptance rate, as in Section 6.3.

Figure 12a and 12b illustrate the trade-off between patient survival and redundant volunteers for both our adaptive and static strategies under a 50% acceptance rate, with 6-minute and 9-minute EMS response times, respectively. The best static policies are *Send 1 at time 0* for a 6-minute EMS response time, and *Send 2 at time 0* under a 9-minute EMS response time. Similar to Fig. 6, the tree-based selection strategies under 6-minute and 9-minute EMS response times are able to improve both patient survival rates and redundant volunteers, with the 6-minute case showing a more substantial increases in patient survival. This is possibly due to the fact that, if the tree-based strategy chooses to send more alerts than the best static strategy in the 6-minute case, this suggests that there are multiple volunteers located very close to the patient and that the high acceptance rate allows these nearby volunteers to substantially improve patient survival.

Under a higher volunteer acceptance rate, the current SJAS strategy now comes very close to the frontier. This is likely because now we can get volunteer acceptances from the first few batches with high probability, avoiding the need to send additional alerts later in the process, which previously incurred more redundant volunteers.

Figure 13 shows the percentage improvement in patient survival versus the percentage improvement in the objective of the tree-selected policy compared to the best static policy for each test case under a 50% acceptance rate and a 6-minute EMS response time. The best static strategy under both 14% and 50% acceptance rates with a 6-minute EMS response time is *Send 1 at time 0*. Similar to Fig. 11, and compared to Fig. 7, the tree-based strategy under a 6-minute EMS response time improves upon the best static strategy in most cases by increasing patient survival, rather than saving on redundant volunteers. However, compared to Fig. 11, the cluster of red points in the lower left corner of Fig. 13, where the tree-based strategy recognizes that a CFR response is unnecessary, shows a more significant improvement in the overall objective because the high acceptance rate leads to a higher probability of having redundant volunteers on-scene.

## Appendix C. Automated external defibrillators

In this appendix, we present a case study that incorporates automated external defibrillators (AEDs). Our goal is to demonstrate the feasibility of training tree-based policies in this more complex setting, not to provide a definitive statement about policies that account for AED availability. We begin by describing how AED locations are generated for each OHCA incident in Appendix C.1, followed by an explanation of how we model volunteer dispatch when publicly available AEDs are included in Appendix C.2. We introduce a different survival function and a different definition of redundant volunteers for performance evaluation in Appendix C.3. Finally, Appendix C.4 presents the corresponding numerical results.

### Appendix C.1. AED locations

There are approximately 3000 publicly available AEDs in the portion of Auckland we study [10]. Without more detailed AED location data, we assume that all 287 area units within our study region, covering a total of 499 square kilometers, share a uniform AED density. Under this assumption, an average of 19 AEDs are expected to fall within the dispatch radius of each OHCA incident.

For each simulated OHCA incident, we first sample the number of AEDs within the dispatch radius from a Poisson distribution with the above AED density. We then generate the spatial locations of these AEDs by sampling uniformly within the dispatch radius centered at the patient location. Given the volunteers’ locations, we compute for each volunteer the shortest detour distance required to retrieve an AED before continuing to the patient. These detour distances, together with the volunteers’ direct travel distances, are the inputs to our discrete-event simulation model that includes AEDs.

### Appendix C.2.Dispatching volunteers with AEDs

We follow the same CFR response process described in Fig. 1. However, now every alerted volunteer is informed of both the location of the OHCA and the AED location, specific to that volunteer, that yields the shortest detour en-route to the OHCA.

When a volunteer accepts an alert, they decide whether to pick up an AED before proceeding to the patient. We assume that each accepting volunteer goes directly to the patient with an incident- and volunteer-specific probability *p*, and with probability $1-p$ first detours to retrieve the AED and then continues to the OHCA. This probability *p* depends on the relative travel times of the two options. Let $t_{direct}$ denote the travel time if the volunteer goes directly to the OHCA, and $t_{divert}$ the travel time when diverting to pick up the AED. We define

9 $$\begin{aligned} p = 1 - \frac{t_{direct}}{t_{divert}}, \end{aligned}$$

so that volunteers are more likely to go directly to the OHCA when doing so is substantially faster. For simplicity, we assume that each accepting volunteer independently decides whether to divert or not.

Under this framework, the set of pre-defined policies introduced in Section 6.1 can be extended naturally to the AED setting. The difference is that instead of alerting volunteers based on proximity, now we prioritize those with the shortest AED-detour travel time, since AEDs provide a higher chance of patient survival compared to CPR alone. In addition, alerting no longer stops upon first acceptance. Instead, we stop sending out further alerts once a volunteer accepts *and* chooses to pick up an AED along the way.

### Appendix C.3.Performance metrics

Now that volunteer responses include both CPR-only and AED interventions, we can no longer rely on the original survival function from [36] used in the main paper. Volunteers who pick up an AED en route can provide more effective assistance than CPR-only responders but still cannot fully match the level of care given by EMS.

To quantify patient survival in this extended setting, we follow [16], which models patient survival as a function of three key time points: the earliest arrival of any responder, whether a CPR-only volunteer, an AED-equipped volunteer, or EMS, which we call the *time to CPR*, the earliest arrival of any responder capable of delivering a shock, whether an AED-equipped volunteer or EMS, which we call *the time to AED*, and the time to EMS arrival. All times are measured in minutes from the moment of OHCA onset. The survival function is given by

10 $$\begin{aligned} \phi (t_{CPR}, t_{AED}, t_{EMS}) = \max (0, 0.67 - 0.023 \\ \times t_{CPR} - 0.011 \times t_{AED} - 0.021 \times t_{EMS}) \end{aligned}$$

In addition, we must also redefine redundant volunteers in the AED setting. If the first responder provides only CPR, then a subsequent volunteer arriving with an AED can still deliver additional, more effective care and therefore should not be classified as redundant. If the first responder is a CPR-only volunteer, then among all subsequent volunteer arrivals, only the *first* AED-equipped volunteer who arrives before EMS is considered non-redundant. If the first responder is capable of delivering a shock, whether an AED-equipped volunteer or EMS, then all volunteers arriving afterward are considered redundant.

### Appendix C.4. Numerical Results

Following the machine learning pipeline described in Section 4.3, we generated 10,000 instances as our training set and labeled each instance with its optimal dispatch policy using our discrete simulation model with the new performance metrics introduced above. We also generated 2,000 instances as our testing set. In addition to the predictors used in the original classification tree included in Section 4.3, we now incorporate each volunteer’s AED response time, defined as the sum of the realized triage delay and the volunteer’s shortest AED-detour time, to reflect the inclusion of AED in this extended setting.

Figure 14 presents the tree-based selection strategy for AED-incorporated instances in Auckland under a 14% acceptance rate and a 12-minute EMS response time. The resulting tree-based strategy remains highly interpretable in this extended setting. However, compared with Figs. 3 and  9, the tree in Fig. 14 is more complex and selects a broader range of dispatch policies. This increased complexity likely arises from the use of the different survival function. Under our evaluation of survival rate using [16], patient survival is zero when no volunteer arrives before EMS. Therefore, sending alerts to as many volunteers as possible is favored, as it increases the likelihood that a volunteer, whether with an AED or not, arrives early enough to provide a positive survival benefit that outweighs the cost of redundant volunteers, and the count of redundant volunteers is limited due to the low acceptance rate.

In addition, Figure 14 shows that volunteers’ AED response times are more significant predictors, as many of the tree’s splits occur on these variables. This is expected, since volunteer responses with AEDs provide greater survival benefits than CPR-only responses. Direct response time becomes more significant when a volunteer’s direct travel time to the patient is substantially smaller than their AED-detour travel time. Even if such a volunteer is unlikely to arrive with an AED, they may still reach the scene early enough to provide CPR, which remains valuable in this setting where the absence of any volunteer response results in zero survival probability.

Figure 15 illustrates the trade-off between patient survival and redundant volunteers for both the tree-based and static strategies with AEDs incorporated, under a 14% acceptance rate and a 12-minute EMS response time. As shown in the figure, the current SJAS strategy still lies below the frontier, giving lower patient survival and more redundant volunteers. Our tree-based selection strategy, on the other hand, lies far outside the efficient frontier of the static strategy, achieving comparable patient survival rates while requiring fewer redundant volunteers than the best static strategy, which in this case is *Send 7 at time 0*.

It is worth noting that the classification accuracy of the tree-based strategy drops to 0.76 in the AED-incorporated setting, likely because the introduction of additional predictors and a broader set of feasible policies increases the complexity of the decision space. This observation suggests that our tree-based approach could become even more effective with improvements in model accuracy or the design of a more refined set of pre-defined dispatch policies.

## Appendix D. Sensitivity analysis on the penalization factor

Here we explore how the choice of $\gamma$ influences patient survival and redundant volunteers. As defined in Section 3, the $\gamma$ factor balances between maximizing patient survival and minimizing redundant volunteers. A larger value of $\gamma$ corresponds to a higher penalty for redundant volunteers, suggesting that a high level of frustration from being redundant on-scene may negatively affect future participation and, ultimately future survival outcomes.

To assess the impact of $\gamma$, we use the same training and testing sets of Auckland OHCA instances, under a 14% acceptance rate and a 12-minutes EMS response, as in Section 6.1. We then fit a tree-based selection strategy across a range of different $\gamma$ values. Figure 16 shows the resulting trade-off between patient survival and the number of redundant volunteers for the tree-based selection strategy under each value of $\gamma$.

As seen in Fig. 16, large values of $\gamma$ lead to lower expected survival and fewer redundant volunteers. This occurs because higher penalization discourages sending many alerts, hence reducing the likelihood of redundant volunteers. This is also reflected in the best static strategies selected under different $\gamma$ values: with $\gamma =0.001$, the best static strategy is *Send 10 at time 0*, whereas with $\gamma = 0.05$, the best static strategy is *Send 1 at time 0*. Ultimately, the appropriate choice of $\gamma$ depends on the service provider’s tolerance for redundant volunteers and their understanding of how such redundancy affects volunteer engagement and long-term system performance.

It is also worth noting, though not shown in the figure, that *Keep n active until time t* policies are more likely to appear in the tree-based policy when $\gamma$ is large. This likely arises because, under a high penalization factor, it becomes better to wait for initial alerted volunteers to respond before sending additional alerts, especially in settings with a relatively long EMS response time of 12 minutes.
